# The γ-tubulin-specific inhibitor gatastatin reveals temporal requirements of microtubule nucleation during the cell cycle

**DOI:** 10.1038/ncomms9722

**Published:** 2015-10-27

**Authors:** Takumi Chinen, Peng Liu, Shuya Shioda, Judith Pagel, Berati Cerikan, Tien-chen Lin, Oliver Gruss, Yoshiki Hayashi, Haruka Takeno, Tomohiro Shima, Yasushi Okada, Ichiro Hayakawa, Yoshio Hayashi, Hideo Kigoshi, Takeo Usui, Elmar Schiebel

**Affiliations:** 1 Graduate School of Life and Environmental Sciences, University of Tsukuba, 1-1-1 Tennodai, Tsukuba 305-8572, Japan; 2 Zentrum für Molekulare Biologie der Universität Heidelberg, ZMBH-DKFZ Alliance, Im Neuenheimer Feld 282, Heidelberg 69120, Germany; 3 Graduate School of Pure and Applied Sciences, University of Tsukuba, 1-1-1 Tennodai, Tsukuba 305-8571, Japan; 4 Department of Medicinal Chemistry, Tokyo University of Pharmacy and Life Sciences, Hachioji, Tokyo 192-0392, Japan; 5 Laboratory for Cell Polarity Regulation, RIKEN Quantitative Biology Center, Suita 565-0874, Japan; 6 Division of Applied Chemistry, Graduate School of Natural Science and Technology, Okayama University, 3-1-1 Tsushima-naka, Kita-ku, Okayama 700-8530, Japan

## Abstract

Inhibitors of microtubule (MT) assembly or dynamics that target α/β-tubulin are widely exploited in cancer therapy and biological research. However, specific inhibitors of the MT nucleator γ-tubulin that would allow testing temporal functions of γ-tubulin during the cell cycle are yet to be identified. By evolving β-tubulin-binding drugs we now find that the glaziovianin A derivative gatastatin is a γ-tubulin-specific inhibitor. Gatastatin decreased interphase MT dynamics of human cells without affecting MT number. Gatastatin inhibited assembly of the mitotic spindle in prometaphase. Addition of gatastatin to preformed metaphase spindles altered MT dynamics, reduced the number of growing MTs and shortened spindle length. Furthermore, gatastatin prolonged anaphase duration by affecting anaphase spindle structure, indicating the continuous requirement of MT nucleation during mitosis. Thus, gatastatin facilitates the dissection of the role of γ-tubulin during the cell cycle and reveals the sustained role of γ-tubulin.

## Abstract (web summary)

Current microtubule inhibitors target α/β-tubulin, but no specific inhibitor of γ-tubulin has been developed. Here the authors present gatastatin as a γ-tubulin inhibitor and use it to probe the role of γ-tubulin during the cell cycle.

## Graphical abstract

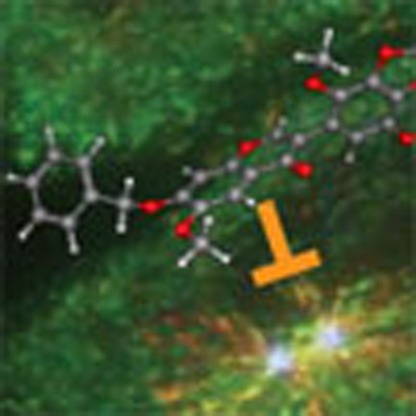

Microtubules (MTs) are dynamic polymers of α/β-tubulin heterodimers that are involved in a wide variety of biological functions such as mitosis, organelle positioning and cell motility. MTs are inherently polar structures with α-tubulin terminating the MT minus end and β-tubulin the MT plus end. While α/β-tubulin heterodimers can spontaneously polymerize to generate MTs *in vitro*, MT nucleation *in vivo* is initiated from a ring-like template of γ-tubulin (another member of the tubulin superfamily) that can promote MT nucleation at concentrations below those required for spontaneous assembly^1^,^2^,^3^. γ-Tubulin recruits accessory proteins, so-called γ-tubulin complex proteins (GCPs). γ-Tubulin, GCP2 (ref. 4) and GCP3 (ref. 5) form a tetrameric 2:1:1 complex named the small γ-tubulin complex (γ-TuSC). In many eukaryotes, γ-TuSC assembles with additional GCPs (GCP4–6) into the stable γ-tubulin ring complex (γ-TuRC)^6^. Despite the importance of γ-tubulin function for MT formation, γ-tubulin-specific MT nucleation inhibitors are yet to be reported. This deficiency in our drug repertoire limits the temporal analysis of γ-tubulin functions in eukaryotic cells to lengthy short interfering RNA (siRNA) depletion experiments that arrest cells in prometaphase because of spindle assembly checkpoint (SAC) activation after sustained deficiency in γ-tubulin functions for many hours before observation. We therefore lack a clear understanding of the requirements of γ-tubulin at discrete cell cycle phases that arises from acute inhibition of γ-tubulin functions through pharmacological intervention.

Here we used recombinant human γ-tubulin to screen for γ-tubulin inhibitors and identified the AG1 (refs 7, 8) derivative gatastatin^9^ as γ-tubulin-specific inhibitor. Gatastatin blocked γ-tubulin-dependent MT nucleation, without affecting α/β-tubulin polymerization. Gatastatin identified novel γ-tubulin functions for metaphase spindle maintenance and anaphase spindle elongation. These data demonstrate the continuous importance of γ-tubulin throughout the cell cycle for MT homeostasis.

## Results

### Screening of γ-tubulin binders from α/β-tubulin inhibitors

γ-Tubulin shares 34% similarity with β-tubulin (UniProtKB/Swiss-Prot: P23258.2 and Q13509.2). This prompted us to ask whether it would be possible to develop γ-tubulin-specific inhibitors from known drugs that bind to the colchicine-binding site in β-tubulin, for example, nocodazole, plinabulin^10^ and glaziovianin A^7^,^8^ (AG1). We screened a collection of β-tubulin colchicine-site binders for binding to human γ-tubulin (Table 1 and Supplementary Fig. 1). The correct folding of the purified, recombinant γ-tubulin was confirmed by two criteria. First, γ-tubulin bound α-[^32^P]-GTP with high affinity^11^ (Supplementary Fig. 2a). Second, purified human γ-tubulin and GCP4 assembled into a stable complex^12^ (Supplementary Fig. 2b).

Changes in tryptophan fluorescence of γ-tubulin were used as a test for drug binding^13^. Nocodazole and plinabulin both bound γ-tubulin, however, with a markedly lower affinity than for α/β-tubulin (*K*_d_[α/β-tubulin]/*K*_d_[γ-tubulin]<0.05; Table 1). In contrast, AG1 bound γ-tubulin (*K*_d_=85.3 μM, Table 1) and α/β-tubulin with similar *K*_d_ values (*K*_d_[α/β-tubulin]/*K*_d_[γ-tubulin]=0.61). Thus, AG1 binds to both γ-tubulin and α/β-tubulin.

**Figure 1:**
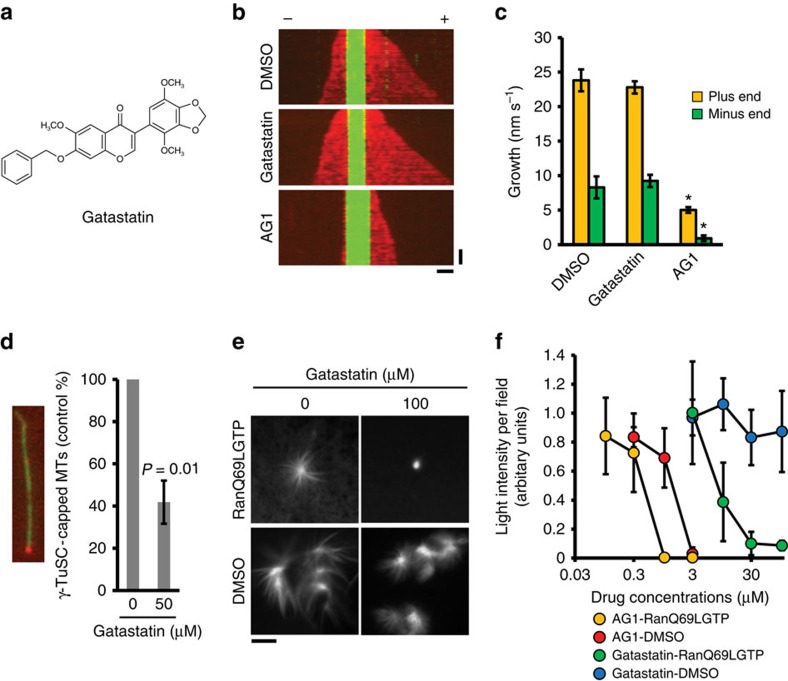
Gatastatin is a γ-tubulin-specific inhibitor. (**a**) The chemical structure of gatastatin. (**b**) The effect of gatastatin on MT dynamics *in vitro* was monitored as described in the Methods section. Kymographs of Alexa647-labelled MT polymerization (red) from tetramethylrhodamine- and biotin-labelled GMPCPP MT seeds (green) in the presence of GTP and either 1% DMSO, 30 μM gatastatin or 30 μM AG1. Horizontal scale bar, 2 μm; vertical scale bar, 1 min. (**c**) Quantification of the compound's impact on the velocity of MT growth. Data are average velocities±s.e.m. calculated from 31 MTs (plus end) and 28 MTs (minus end) for DMSO, 23 MTs (plus end) and 22 MTs (minus end) for gatastatin, 23 MTs (plus end) and 20 MTs (minus end) for AG1. One-way ANOVA with Tukey's multiple comparisons test was used to determine the significance of the difference using the GraphPad Prizm 6 software. *P<0.0001. There is no significant difference between the DMSO and gatastatin samples (*P*>0.05). (**d**) The effect of gatastatin on the binding activity of γ-TuSC to paclitaxel-stabilized MTs. γ-TuSC was treated with 50 μM of gatastatin and was incubated with paclitaxel-stabilized MTs. MTs and γ-TuSC were visualized by immunofluorescence on the coverslips using anti-α-tubulin (green) and anti-His tag (red, γ-TuSC) antibodies, respectively. At least 440 MTs were counted in each experiment. Three independent experiments were performed. Error bars represent s.d. Two-tailed, paired Student's *t*-test was used to obtain *P* value. (**e**) The effects of gatastatin on the RanQ69L- and DMSO-stimulated aster formation. Egg extracts with Cy3-tubulin and gatastatin were incubated for 20 min at 20 °C in the presence of RanQ69L or 5% DMSO. Aster formation was analysed by fluorescence microscopy. Scale bar, 5 μm. (**f**) The average light intensity of asters in at least 10 randomly selected fields with a × 10 objective was quantified. Three independent experiments were performed. Error bars represent s.d.

To identify molecules that showed selectivity for γ-tubulin over α/β-tubulin, we synthesized derivatives of both plinabulin and AG1 and tested their binding affinity for γ-tubulin and α/β-tubulin. KPU-406, one of the plinabulin derivatives (Supplementary Fig. 1), bound to γ-tubulin (*K*_d_=46.1 μM, Table 1) and α/β-tubulin with similar *K*_d_ values (*K*_d_[α/β-tubulin]/*K*_d_[γ-tubulin]=0.57). Interestingly, the AG1 derivative gatastatin (Fig. 1a) had a markedly 12-fold higher affinity for γ-tubulin (*K*_d_=3.6 μM, Table 1) than for α/β-tubulin (*K*_d_[α/β-tubulin]/*K*_d_[γ-tubulin]=11.81). These data suggest that gatastatin binds relatively specific to γ-tubulin.

### Gatastatin is a γ-tubulin-specific inhibitor

We next investigated the impact of gatastatin on MTs assembled from purified tubulin in the absence of γ-tubulin. In sharp contrast to AG1, which inhibits dynamic behaviour of MTs and therefore reduces MT polymerization^8^, gatastatin failed to impair MT polymerization *in vitro* when this polymerization had been induced by either addition of glutamate^10^, paclitaxel or recombinant Tau protein (Supplementary Fig. 3a–c). Moreover, gatastatin did not affect MT growth velocity as measured by total internal reflection fluorescence microscopy (TIRF) analysis of single MTs (Fig. 1b,c). In contrast, gatastatin blocked γ-TuSC-stimulated MT polymerization (Supplementary Fig. 3d) at the same concentration, as it was ineffective in blocking glutamate, paclitaxel and Tau-induced MT formation or affecting MT dynamics. Thus, gatastatin only affects γ-tubulin-dependent MT polymerization.

We previously reported that GTP binding of γ-tubulin is important for MT nucleation and viability of yeast cells^14^. We therefore tested the effect of gatastatin on GTP-binding activity of γ-tubulin in a α-[^32^P]-GTP crosslinking assay. Gatastatin inhibited GTP binding to human γ-tubulin (Table 2). Using the same assay, we showed that at an AG1 concentration that inhibits MT polymerization (Fig. 1b,c and Supplementary Fig. 3a–c), it failed to affect GTP-binding to γ-tubulin (Table 2). Because gatastatin did not affect paclitaxel-stimulated MT polymerization (Supplementary Fig. 3b), we also tested the impact of the drug on the binding of γ-TuSC to stabilize MTs. As shown in Fig. 1d, gatastatin impaired γ-TuSC binding to MT ends. Together, these data indicate that gatastatin is a specific inhibitor for γ-tubulin-dependent MT nucleation, while AG1 affects α/β-tubulin without influencing γ-tubulin-stimulated MT formation.

To confirm the specificity of gatastatin towards γ-tubulin-dependent MT nucleation in a more physiological environment, we analysed MT formation in M-phase-arrested *Xenopus* egg extracts. Gatastatin strongly attenuated γ-tubulin-dependent, RanQ69L-stimulated aster formation (IC_50_[RanQ69L-aster]=9.7 μM, Fig. 1e,f), but had no influence on dimethylsulphoxide (DMSO)-stimulated, γ-tubulin-independent aster formation (IC_50_[DMSO-aster]=>100 μM, Fig. 1e,f). Quantitative analysis revealed that gatastatin reduced the radius and the MT density of RanQ69L-dependent asters without changing the number of asters (Fig. 1f and Supplementary Fig. 4d,e). Moreover, γ-tubulin-dependent, centrosome-induced aster formation was nearly completely inhibited by gatastatin (Supplementary Fig. 4f). In contrast, AG1 reduced the radius and MT density of both γ-tubulin-dependent (RanQ69L- and centrosome-induced) and γ-tubulin-independent (DMSO-induced) MT polymerization (IC_50_[RanQ69L-aster]=0.44 μM, IC_50_[DMSO-aster]=1.37 μM, Fig. 1f and Supplementary Fig. 4c–e,g). The ratio of the half-maximal inhibitory concentration (IC_50_) values confirmed that gatastatin specifically inhibits γ-tubulin-induced aster formation while AG1 is a general tubulin polymerization inhibitor (Fig. 1f; IC_50_[DMSO-aster]/IC_50_[RanQ69L-aster]=>10.4 and 3.14, respectively). In conclusion, gatastatin specifically inhibits γ-tubulin-induced MT assembly.

### Gatastatin inhibits MT nucleation activity of centrosomes

**Figure 2:**
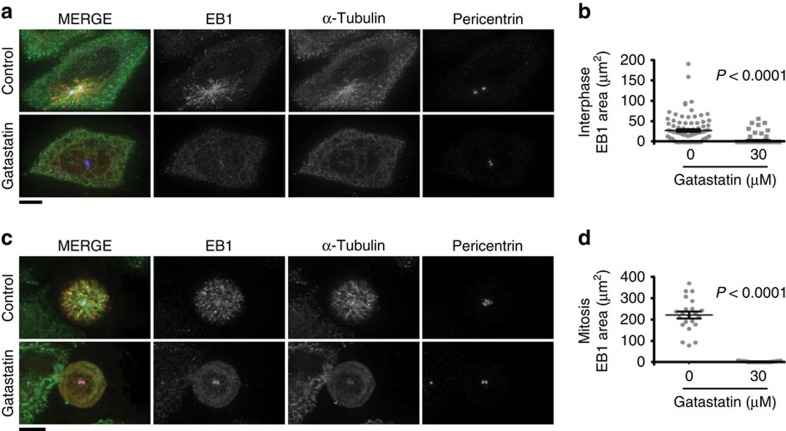
Impact of gatastatin on MT regrowth in HeLa cells. (**a**) The impact of gatastatin on MT nucleation by centrosomes in interphase. After nocodazole and ice treatments, MT nucleation (1 min at 37 °C) in the presence of 30 μM of gatastatin or the DMSO solvent control was monitored. Red, green and blue in the merged image represent EB1, α-tubulin and pericentrin, respectively. Scale bar, 10 μm. Three independent experiments were performed and one representative experiment is shown. (**b**) The area of EB1 around centrosomes in **a** was calculated using the ImageJ software from 97 (control) or 84 (gatastatin) centrosomes. Error bars represent s.e.m. Two-tailed, unpaired Student's *t*-test was used to obtain *P* value. (**c**) The impact of gatastatin on MT nucleation by centrosomes in mitosis. After STLC and ice treatments, spindle MT nucleation (1 min at 20 °C) in the presence of 30 μM of gatastatin or the DMSO solvent control was monitored. Red, green and blue in merged image represent EB1, α-tubulin and pericentrin, respectively. Scale bar, 10 μm. Three independent experiments were performed and one representative experiment is shown. (**d**) The area of EB1 around centrosomes in **c** was calculated using ImageJ software from 22 (control) or 17 (gatastatin) monopolar spindles. Error bars represent s.e.m. Two-tailed, unpaired Student's *t*-test was used to obtain *P* value.

γ-Tubulin localizes at centrosomes where it facilitates MT nucleation in interphase and mitosis^6^,^15^. To estimate whether gatastatin has the potential to inhibit γ-tubulin in human cells, we analysed the effect of gatastatin on γ-tubulin-dependent centrosomal MT nucleation activity. For this analysis, the MT network was first depolymerized either by nocodazole and cold treatment (interphase) or only cold treatment (mitosis). Subsequently, centrosome-organized MTs were allowed to re-grow by returning cells to permissive conditions. For MT regrowth analysis, we detected the plus TIP protein EB1 with antibodies. EB1 associates with growing MT plus tips^16^. On nocodazole washout/warming up of the cells, the EB1 and tubulin signals developed around interphase and mitotic centrosomes in the control cells (Fig. 2a,c). This centrosome MT nucleation activity was γ-tubulin-dependent as it was reduced in cells with siRNA-depleted γ-tubulin (Supplementary Fig. 5a–f). Importantly, gatastatin inhibited MT nucleation activity of interphase and mitotic centrosomes of HeLa cells (Fig. 2a–d). Similar inhibition of γ-tubulin's MT nucleation activity by gatastatin was observed in RPE-1 and U2OS cells (Supplementary Figs 6 and 7). Interestingly, in both interphase and mitotic cells, γ-tubulin localization to centrosomes was not affected by gatastatin (Supplementary Fig. 8). Taken together, these results strongly suggest that gatastatin inhibits γ-tubulin-dependent MT nucleation of centrosomes in human cells without affecting γ-tubulin localization.

### Gatastatin alters MT dynamics of interphase cells

**Figure 3:**
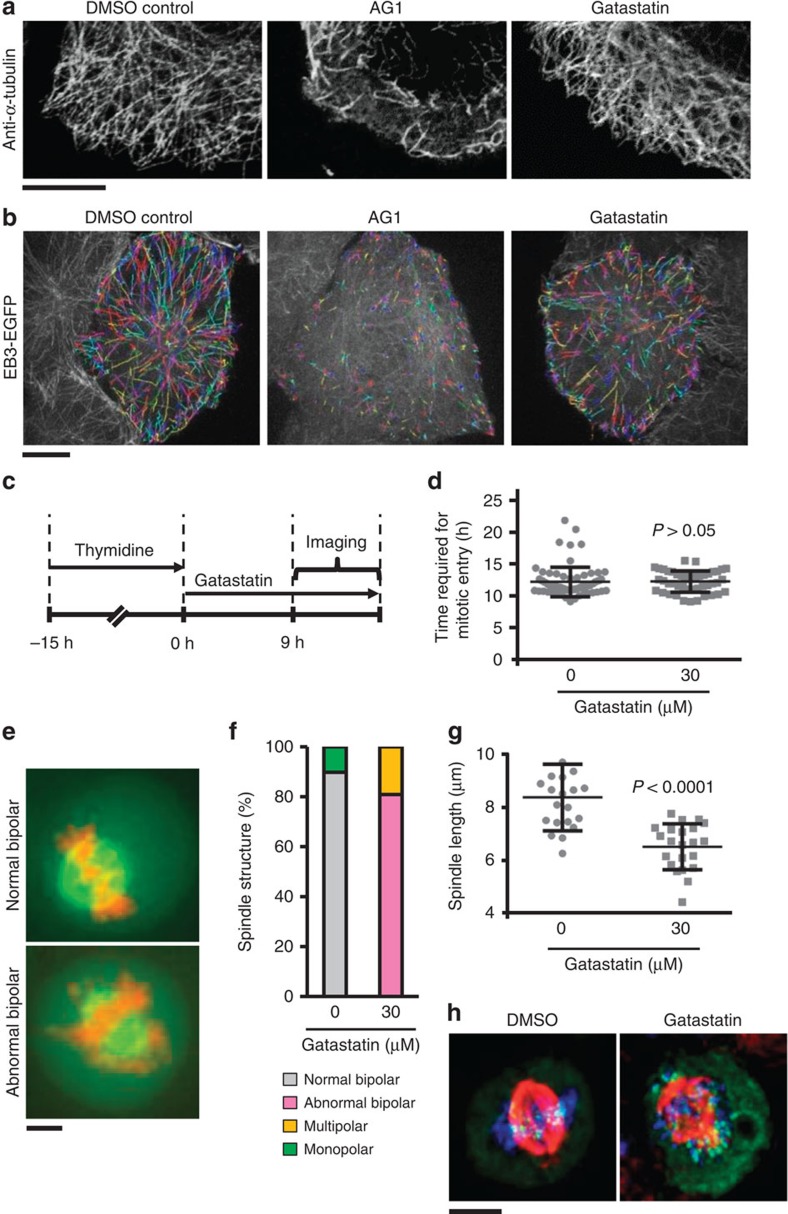
Effects of gatastatin on interphase MT network, interphase MTs dynamics, mitotic entry and mitotic spindle formation. (**a**) The effect of gatastatin on the interphase MT network. HeLa cells were treated with 1% DMSO, 30 μM AG1 or 100 μM gatastatin for 2.5 h. The MT network was observed using a LSM 700 laser-scanning confocal microscope. Scale bar, 10 μm. (**b**) The effects of AG1 and gatastatin on MT dynamics. HeLa cells expressing EB3-EGFP were treated with DMSO, 3 μM AG1 or 30 μM gatastatin for 15 min. EB3 tracks are displayed in rainbow colours as maximum intensity projection of all time points from a 60-s sequence of imaging. Scale bar, 10 μm. MT parameters are summarized in Table 3. (**c**,**d**) γ-Tubulin function is not required for the cell cycle progression from S phase to mitotic entry. (**c**) Scheme of the experiment in **d**. After HeLa cells were arrested at S phase with 2 mM thymidine for 15 h, thymidine was removed and the timings of nuclear envelope breakdown and chromosome condensation in the presence or absence of 30 μM gatastatin were observed by time-lase microscopy. (**d**) The time required for mitotic entry was calculated. Error bars represent s.d. Two-tailed, unpaired Student's *t*-test was used to obtain *P* value. (**e**) The effect of gatastatin on the mitotic spindle structure of cells from the experimental set-up of **c**. Green and orange represent α-tubulin and DNA, respectively. Scale bar, 5 μm. (**f**) Spindles in **e** were analysed from 39 (control) or 47 (gatastatin) cells. (**g**) Gatastatin reduces spindle length. The distance between two pericentrin signals was measured as the length of the mitotic spindle in the presence or absence of 30 μM gatastatin (spindle length=
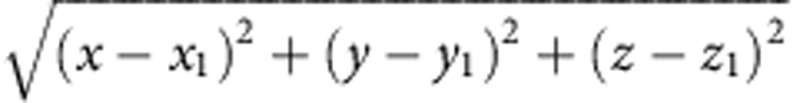
). The length of 20 spindles per experiment was analysed. Three independent experiments were performed and one representative experiment is shown. Error bars represent s.d. Two-tailed, unpaired Student's *t*-test was used to obtain *P* value. (**h**) BubR1 localized on misaligned chromosome around poles in 30 μM gatastatin-treated cells. Green, red and blue represent BubR1, α-tubulin and DNA, respectively. Scale bar, 5 μm.

Conditional lethal γ-tubulin mutations or siRNA depletion of γ-tubulin complex components affect MT dynamics and spindle assembly in yeast and *Drosophila* cells^14^,^17^,^18^,^19^,^20^. However, because siRNA depletion is slow, it is unclear whether this impact on MT dynamic changes is a direct consequence of γ-tubulin depletion. Furthermore, SAC activation by γ-tubulin depletion arrests cell cycle progression in prometaphase, making it difficult to draw firm conclusions about γ-tubulin functions in other mitotic phases. Gatastatin overcomes these limitations because of its rapid inhibition of γ-tubulin. We first ask whether a block to γ-tubulin function alters interphase MT dynamics. Gatastatin (100 μM) addition did not obviously change the appearance of the interphase MT network in HeLa cells. The MT density was similar in DMSO control and 100 μM gatastatin-treated cells 2.5 h after drug addition (Fig. 3a). In contrast, addition of 30 μM AG1 that binds to β-tubulin and inhibits MT dynamics^8^ caused partial depolymerization of interphase MT network (Fig. 3a).

We next tracked EGFP-labelled EB3 to measure MT dynamics in interphase cells. EB3 is a plus TIP protein that specifically associates with the growing plus ends of MTs^16^. Gatastatin and AG1 (ref. 8) both decreased the average speed and track length of EB3 signal 15 min after drug addition to cells (Fig. 3b and Table 3). However, in contrast to the clear reduction of the lifetime of EB3 at MT plus ends following AG1 treatment, gatastatin increased the lifetime of the EB3 signals (Table 3). Moreover, gatastatin did not affect the number of EB3 tracks. Thus, γ-tubulin inhibition by gatastatin reduces MT dynamics without affecting the number of growing MTs.

### Gatastatin inhibits spindle formation but not mitotic entry

We analysed the consequences of gatastatin treatment on mitotic entry and spindle formation. HeLa cells were synchronized in the S phase followed by treatment with either gatastatin or the solvent DMSO. The timing of mitotic entry was virtually identical in both cases (Fig. 3c,d). However, gatastatin impaired spindle formation as ∼80% of mitotic cells showed abnormal bipolar spindles with misaligned chromosomes (Fig. 3e). α-Tubulin density was not markedly affected in these cells (Supplementary Fig. 9). Furthermore, the bipolar spindles of gatastatin-treated cells were shorter than those of control cells (Fig. 3g), and the SAC remained active as indicated by the BubR1 signal on kinetochores of misaligned chromosomes (Fig. 3h). Thus, γ-tubulin activity is needed for bipolar spindle formation.

### Gatastatin reduces the length of metaphase spindles

**Figure 4:**
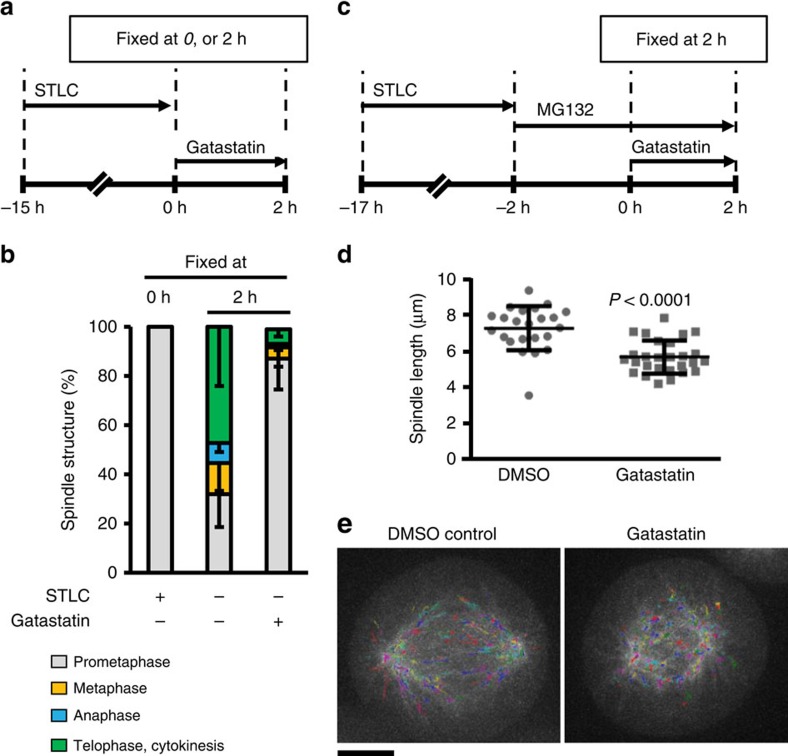
Impact of gatastatin on mitotic spindle dynamics. (**a**,**b**) γ-Tubulin function is important for normal bipolar spindle formation. (**a**) Scheme of the experiment in **b**. HeLa cells were arrested at prometaphase by treatment with 10 μM of STLC for 15 h before STLC was washed out. Progression through mitotic phases was then monitored either in the presence or absence of 30 μM of gatastatin for 2 h. (**b**) The mitotic stages of 20 cells per experiment from the scheme in **a** were analysed in three independent experiments. Error bars represent s.d. (**c**–**e**) γ-Tubulin function is important for normal bipolar spindle maintenance. (**c**) Scheme of the experiment in **d**,**e**. After STLC washout, HeLa cells were arrested at metaphase with bipolar spindles by treatment with 5 μM of MG132 for 2 h. 30 μM Gatastatin was added to cells and incubated for 2 h in the presence of MG132. (**d**) The spindle length (distance between centrosomes) of cells in scheme **c** was measured as described in Fig. 3g. Spindles of 20 cells per experiment were analysed. Three independent experiments were performed and one representative experiment is shown. Error bars represent s.d. Two-tailed, unpaired Student's *t*-test was used to obtain *P* value. (**e**) The effect of gatastatin on spindle MT dynamics was analysed using HeLa cells expressing EB3-EGFP. EB3 signals of spindle in **c** were monitored. EB3 tracks are displayed in rainbow colours as maximum intensity projection of all time points from a 60-s sequence of imaging. Scale bar, 5 μm. MT parameters are summarized in Table 4.

An increase in γ-tubulin-dependent MT nucleation activity is required for spindle formation at the beginning of mitosis^21^. Whether γ-tubulin plays a role in mitosis beyond this step is, however, unclear. To assess the functions of γ-tubulin during mitosis, we arrested HeLa cells at defined mitotic phases, followed by addition of gatastatin. We first arrested cells in prometaphase using the reversible Eg5 inhibitor STLC followed by the addition of gatastatin and STLC washout (Fig. 4a). In the presence of gatastatin, 87% of cells were unable to assemble a metaphase-like spindle on STLC washout (Fig. 4b). In contrast, 55% of control cells progressed through metaphase into anaphase, telophase and cytokinesis (Fig. 4b). Thus, γ-tubulin activity is required for the assembly of the metaphase spindle.

Next, we tested the impact of gatastatin on preformed metaphase-like spindles that were generated by MG132 inhibition of proteasome function after STLC washout (Fig. 4c–e)^22^. Gatastatin did not lead to metaphase spindle collapse (Supplementary Fig. 10). However, gatastatin reduced the length of the metaphase spindle by 22% compared with the control (Fig. 4d). In addition, imaging HeLa EB3-EGFP showed that gatastatin reduced the number of growing MTs by 17% (Fig. 4e and Table 4). We conclude that γ-tubulin activity is continually required for both spindle assembly in prometaphase and for maintaining the length and dynamicity of the metaphase spindle.

### Gatastatin prolongs anaphase duration

**Figure 5:**
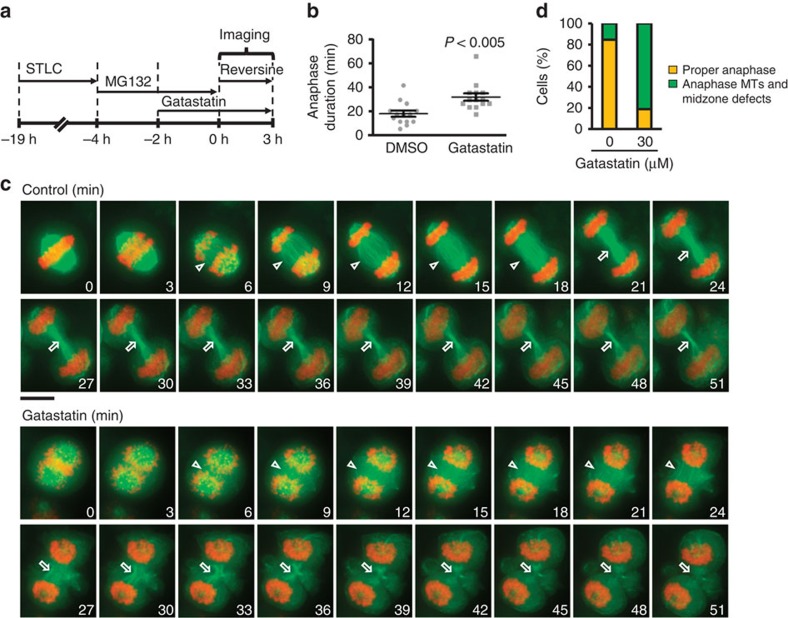
Impact of gatastatin on anaphase spindle elongation. γ-Tubulin function is important for the anaphase progression. (**a**) Scheme of the experiment in **b**–**d**. After washout of MG132 from 30 μM gatastatin-treated metaphase cells, the transition into anaphase, telophase or cytokinesis was observed in the presence of reversine. (**b**) Anaphase duration in **a** was analysed from 14 cells with a × 40 objective. Error bars represent s.e.m. Two-tailed, unpaired Student's *t*-test was used to obtain *P* value. Three independent experiments were performed and one representative data set is shown. (**c**) Anaphase spindle and midzone structure of HeLa cells expressing mCherry-H2B and EGFP-α-tubulin in the presence of 1% DMSO or 30 μM gatastatin was observed with a × 60 objective. Green and orange represent α-tubulin and DNA, respectively. Arrowheads and arrows represent anaphase spindle and midzone, respectively. Scale bar, 5 μm. (**d**) The anaphase spindle and midzone structures in **b** were categorized into two patterns as shown in **c**.

We next measured the transit from metaphase to anaphase on MG132 washout with or without γ-tubulin inhibition (Fig. 5a–d). To overcome the inhibitory influence of the SAC that is triggered by altered MT dynamics, we incubated cells with the MPS1 kinase inhibitor reversine (Fig. 5a)^23^. Time-lapse analysis revealed that gatastatin increased anaphase duration from 18.6 to 32.4 min (Fig. 5b). The MTs in the anaphase spindle were less focused than in control cells (Fig. 5c,d). Midzone MT bundles formed in gatastatin-treated cells (Fig. 5c,d, 30–45 min) but were less focused and organized compared with the control. However, this disorganized midzone recruited the cytokinesis marker CEP55 (ref. 24; Supplementary Fig. 11) and promoted actin ring assembly (Supplementary Fig. 12), indicating that it retained functions. Thus, γ-tubulin activity is required not only for metaphase spindle assembly and maintenance but also for anaphase spindle elongation.

## Discussion

Our study has identified gatastatin as the inhibitor of γ-tubulin that blocks GTP binding to γ-tubulin and γ-tubulin's MT nucleation activity. Several lines of evidence support the notion that gatastatin specifically inhibits γ-tubulin without affecting α/β-tubulin. First, gatastatin showed a 12-fold higher affinity towards γ-tubulin in comparison with α/β-tubulin (Table 1). Second, gatastatin did not affect glutamate, paclitaxel or Tau-induced MT assembly *in vitro* while γ-TuSC-induced MT assembly was inhibited (Supplementary Fig. 3). Third, gatastatin did not affect dynamics of MTs *in vitro* (Fig. 1b,c). Forth, gatastatin blocked binding of γ-TuSC to the minus end of preformed MTs (Fig. 1d). Fifth, gatastatin inhibited γ-tubulin-dependent MT nucleation in *Xenopus* egg extracts while DMSO-induced MT assembly was unaffected (Fig. 1e,f). Finally, gatastatin blocked γ-tubulin-dependent MT nucleation activity of centrosomes in human cells (Fig. 2). Together, these experiments provide a strong case for the γ-tubulin specificity of gatatastin. However, we cannot exclude the possibility that gatastatin at higher concentrations affects MTs directly.

What is the mode of action of gatastatin? Gatastatin did not misplace γ-tubulin from centrosomes (Supplementary Fig. 8). Thus, it is unlikely that it inhibits Nedd1-dependent recruitment processes^15^. Since GTP binding to yeast γ-tubulin regulates the interaction of γ-tubulin with α/β-tubulin^14^, we suggest that this critical step in MT nucleation is blocked by gatastatin. However, as for paclitaxel that modifies intrinsic MT properties by targeting tubulin, gatastatin probably modulates γ-tubulin activity without invoking a complete inhibition. This view is supported by the observation that gatastatin did not completely inhibit RanQ69L-induced aster formation (Fig. 1e,f).

When used to unravel novel functions of γ-tubulin during the cell cycle, gatastatin highlights the importance of γ-tubulin in the regulation of MT plus end dynamics during interphase and in preassembled metaphase spindles. Consistent with this observation, changes in MT dynamics in response to γ-TuRC components' depletion or mutations in γ-TuSC components have been reported in *Drosophila* S2 cells, *S. pombe* and budding yeast *S. cerevisiae*^14^,^20^,^25^. How γ-tubulin regulates MT plus end dynamics is still unclear. One attractive possibility is that the γ-tubulin complex at the MT minus end^1^ superimposes a structural change on the MT lattice that is propagated along the length of the MT wall to alter the structure and thus dynamics of the remote plus end.

In preassembled metaphase spindles after γ-tubulin inhibition, the pole–kinetochore and pole–pole MTs were not fully functional, as indicated by the persistent activation of the SAC, the reduced number of growing MTs and the reduced spindle length. Thus, γ-tubulin activity is not only needed to assemble the mitotic spindle as this was already indicated by γ-tubulin siRNA depletion analysis^26^ but also to maintain a functional metaphase spindle. A recent study has shown that γ-TuRC actively contributes to spindle architecture by organizing MT minus ends that originate within the main body of the spindle before being transported to the spindle poles^27^.

γ-Tubulin activity was also important for anaphase spindle elongation (Fig. 5). In the presence of gatastatin, the anaphase spindle was much broader and spindle MTs were less organized into parallel bundles of MTs than in the control. Consistent with these defects, gatastatin increased anaphase duration from 18.6 to 32.4 min. Presently, it is unclear whether this anaphase activity of γ-tubulin resides at centrosomes or is bound to MTs via the augmin complex.

During ingression of the cleavage furrow, the MTs of the central spindle become compacted to form the midbody. In the presence of gatastatin, midbody MTs were less organized but still were able to recruit the cytokinesis marker CEP55 (ref. 24) and to induce actin ring assembly (Supplementary Figs 11 and 12). This observation is consistent with the localization of γ-tubulin to the midbody and the finding that γ-tubulin antibody micro-injection experiments disturbed midbody formation without inhibition of cytokinesis^28^.

γ-Tubulin is overexpressed in glioblastoma cells^29^. Furthermore, elevated MT nucleation activity from centrosomes enhances the invasiveness of cancer cells^30^. Reversing the elevated nucleation capacity of centrosomes through γ-tubulin inhibition may offer one route to reduce the aggressiveness and metastatic potential of the wide range of tumour cells in which centrosomes are amplified. Our proof-of-principle study showing that chemical modifications of known α/β-tubulin inhibitors can switch target specificity towards γ-tubulin is therefore an exciting new avenue for the development of novel cancer therapeutics.

## Methods

### Chemical and regents

Colchicine and nocodazole were purchased from Sigma. Plinabulin and AG1 were synthesized as described^9^,^10^. KPU-406 was synthesized as described in the Supplementary Note 1 and Supplementary Figs 13–15. Gatastatin (*O*^7^-demethylbenzyl AG1) was synthesized by a more efficient synthetic method (Supplementary Note 2). All chemicals were dissolved in DMSO.

### Expression and purification of tubulins and γ-TuSC

The human γ-tubulin-myc-6His was expressed in Sf21 insect cells. In brief, 0.7 × 10^6^ cells ml^−1^ were infected with the γ-tubulin baculovirus. After 48 h, cells were collected by centrifugation at 1,0000*g* for 10 min and flash-frozen in liquid nitrogen and stored at −80 °C. Purification was carried out as described with slight modifications^11^. Cells were disrupted in lysis buffer (50 mM KPO_4_ pH 8.0, 500 mM KCl, 1 mM MgCl_2_, 10% glycerol, 10 μM GTP, 1 mM dithiothreitol (DTT)) supplemented with protease inhibitor cocktail (Roche, #11 873 580 001) and centrifuged at 11,950*g* for 10 min at 4 °C. Imidazole (final 10 mM) was added and the lysate was centrifuged again at 192,800*g* for 30 min at 4 °C. Ni-NTA Agarose (QIAGEN, #1018240) was added to the supernatant and incubated for 1 h at 4 °C. Ni-NTA Agarose was then washed twice with wash buffer 1 (lysis buffer containing 25 mM imidazole) and wash buffer 2 (50 mM MES pH 6.6, 500 mM KCl, 5 mM MgCl_2_, 10% glycerol, 10 μM GTP and 1 mM DTT) containing 25 mM imidazole. γ-Tubulin was eluted with wash buffer 2 containing 200 mM imidazole.

Monomeric γ-tubulin was prepared using a size exclusion column HiLoad 16/60 Superdex 200 pre-equilibrated with gel filtration buffer (50 mM MES pH 6.6, 500 mM KCl, 5 mM MgCl_2_, 1 mM EGTA, 10 μM GTP and 1 mM DTT). The peak fractions representing monomeric γ-tubulin were identified by A_280_ and confirmed using SDS–PAGE. Protein was concentrated using a Vivaspin concentrator (Sartorius) and supplemented with 10% glycerol, following snap-freezing in liquid nitrogen and storage at −80 °C.

γ-TuSC was purified as described^14^,^31^. Briefly, γ-TuSC was expressed in HighFive cells and purified by sequential steps of Ni-NTA (Macherei-Nagel) purification, MonoQ column purification (GE Healthcare) and size exclusion (Superdex 200 column, GE Healthcare)^14^. α/β-Tubulin was purified from porcine brain through four cycles of polymerization and depolymerization using 1 M PIPES buffer (1 M PIPES-KOH, 1 mM EGTA, 1 mM MgCl_2_, pH 6.8) for effective removal of MT-associated proteins^32^.

### Purification of GCP4 and Tau recombinant proteins

For expression and purification of full-length GCP4, pET26(+)-GCP4-His_6_ was transformed into *Escherichia coli* strain BL21-CodonPlus. GCP4 was expressed and purified as described with slight modifications^12^.

Cells were cultured in LB medium containing 50 μg ml^−1^ kanamycin and 30 μg ml^−1^ chloramphenicol at 37 °C until the OD_600_ was ∼0.5. Final concentrations of 0.4 mM isopropyl β-D-thiogalactopyranoside was added and protein expression was induced at 25 °C for 6 h. Cells were harvested by centrifuging at 20,000*g* for 20 min at 4 °C and resuspended in lysis buffer (50 mM sodium phosphate pH 8.0, 300 mM NaCl, 10 mM imidazole, 5% glycerol, 1 mM phenylmethyl sulphonyl fluoride (PMSF) and protease inhibitor cocktail (Roche, #11 873 580 001)). Cells were disrupted by sonication on ice and were centrifuged at 20,000*g* for 40 min at 4 °C. Ni-NTA Agarose (QIAGEN, #1018240) and same volume of phosphate buffer (50 mM sodium phosphate pH 8.0, 150 mM NaCl, 5% glycerol and 2.5 mM DTT) containing 10 mM imidazole were mixed with supernatant and incubated for 1 h at 4 °C. The agarose beads were washed twice with phosphate buffer. GCP4 was eluted with 1 ml of phosphate buffer containing 150 mM imidazole.

Monomeric GCP4 was prepared using the size exclusion column HiLoad 16/60 Superdex 200 pre-equilibrated with gel filtration buffer (20 mM Tris-HCl, pH 8.0, 150 mM NaCl and 2 mM DTT). The peak fractions representing monomeric GCP4 were concentrated using a Vivaspin concentrator (Sartorius), following snap-freezing in liquid nitrogen and storage at −80 °C.

For expression and purification of full-length Tau protein, GST-Tau expression plasmid (pTU499) was constructed. Full-length Tau fragment was amplified from pRK174-Tau^33^ using primers 5′-cgGGATCCatggctgagccccgccaggagt-3′ and 5′-ccGAATTCatcacaaaccctgcttggccag-3′ containing BamHI and EcoRI sites (underlined), respectively. The Tau fragment was digested with BamHI and EcoRI and ligated into BamHI and EcoRI sites of the *E. coli* expressing vector pGEX-6P-2. The constructed plasmid, pTU499, was transformed into *E. coli* strain BL21. Cells were cultured in LB medium containing 100 μg ml^−1^ ampicillin at 37 °C until the OD_600_ was ∼0.4. Final concentrations of 0.5 mM isopropyl β-D-thiogalactopyranoside was added and protein expression was induced at 37 °C for 6 h. Cells were harvested by centrifuging at 20,000*g* for 10 min at 4 °C and were resuspended in lysis buffer (1 × PBS containing protease inhibitors (2 mM PMSF and 5 mM benzamidine) and 0.1% Triton X-100). Cells were disrupted by sonication on ice and were centrifuged at 20,000*g* for 20 min at 4 °C. Glutathione-agarose beads (Amersham Biosciences, Cat# 27-4574-01) and final concentrations of 1 mM ATP were added to supernatant and incubated for 1 h at 4 °C. The agarose beads were washed three times with RB buffer (100 mM MES, pH 6.8, 0.5 mM MgCl_2_ and 1 mM EGTA) containing 1 mM ATP. GST-fusion proteins were eluted with 2 ml of RB buffer containing 30 mM glutathione. Glutathione was removed using 10 DG desalting column (Bio-Rad).

### Tryptophan-based drug-binding assay

Gel filtration buffer for γ-tubulin purification (50 mM MES pH 6.6, 500 mM KCl, 5 mM MgCl_2_, 1 mM EGTA and 1 mM DTT) supplemented with 10% of glycerol was used as an assay buffer. γ-tubulin and α/β-tubulin were diluted in this assay buffer to reach a final protein concentration of 1 μM. A final concentration of 10 μM or 1 mM GTP was added to the samples. Proteins were incubated for 30 min with compounds (final DMSO concentration was 1%; 2% for AG1 and gatastatin). After incubation, tryptophan fluorescence of the protein was monitored at 295 nm (excitation) and the scan range was 310–450 nm (emission) using JASCO Spectrofluorometer FP-6500. Dissociation constants were calculated from fitting curves of decreasing fluorescence (ΔFL) using the GraphPad Prizm software.

### Inhibition of GTP binding of γ-tubulin

For analysis of the effect of drugs on GTP binding of γ-tubulin, 70 nM of purified human γ-tubulin in GTP-binding buffer (50 mM MES, 5 mM MgCl_2_, 1 mM EGTA, pH 6.6) was incubated with or without compounds for 15 min at 20 °C. A final concentration of 100 nM of α-[^32^P]-GTP was added and incubated at 20 °C for 30 min. After incubation, bound α-[^32^P]-GTP was crosslinked to γ-tubulin for 60 s by illumination with a ultraviolet lamp using CL-1000 Ultraviolet Crosslinker. Protein with the crosslinked GTP was separated from unbound nucleotide by TCA precipitation and SDS–PAGE, and ^32^P labelling was measured using a Typhoon FLA-7000 (General Electronic Company). Band intensities were quantified with the ImageJ software.

### Measurement of MT dynamics *in vitro*

Purified α/β-tubulin was labelled with tetramethylrhodamine Succinimidyl Ester (C-1171, Life Technologies), Alexa Fluor 647 NHS Ester (A-20106, Life Technologies) or NHS-LC-biotin (21336, Life Technologies) as described^34^. Briefly, α/β-tubulin was polymerized to form MTs. Then, the MTs were centrifuged, resuspended in labelling buffer containing NHS-conjugated dye or biotin. After the labelling reaction, two cycles of polymerization and depolymerization were performed to obtain active α/β-tubulin.

GMPCPP MT seeds were prepared by copolymerization of non-labelled tubulin, tetramethylrhodamine-labelled tubulin and biotin-labelled tubulin with 0.2 mM GMPCPP to yield the rhodamine and biotin labelling ratio 2% and 3%, respectively. GMPCPP was prepared enzymatically from GMPCP (M3170, Sigma-Aldrich) and nucleotide diphosphate kinase (N0379, Sigma-Aldrich)^35^. The glass chambers for observation were prepared as follows. Surface of cover glasses (C022221S, Matsunami Glass) was cleaned by sonication in 1 N KOH and by plasma treatment (Diener), and then silanized with N-2-(aminoethyl)-3-aminopropyl-triethoxysilane (KBE-603, Shin-Etsu Chemical). The amino-silanized glasses were incubated with 200 mg ml^−1^ NHS-PEG (ME-050-TS, NOF) with and without 1 mg ml^−1^ NHS-PEG-biotin (BI-050-TS, NOF) for 3 h at room temperature to make PEG-biotin-coated and PEG-coated glasses^36^. The PEG-biotin-coated glass and the PEG-coated glass were separated using a 30-μm layer of double sticking tape (5603, Nitto-Denko) to make the flow chamber. The MTs were immobilized on the PEG-biotin-coated glass surface via Neutravidin (31000, Thermo), and then the glass surface was blocked with imaging solution (100 mM PIPES-KOH, 2 mM MgSO_4_, 1 mM EGTA, 1 mM GTP, 1% (w/v) Pluronic F-127, 1 mg ml^−1^ casein, 1 mM D-biotin, 2 mM dithiothreitol, 0.2 mg ml^−1^ glucose oxidase, 40 μg ml^−1^ catalase, 1 mM glucose, 0.05% (v/v) methylcellulose, pH 6.9). Finally, 5 μM tubulin containing 100 nM Alexa Fluor 647-labelled tubulin and 1% (v/v) DMSO, 30 μM Gatastatin or 30 μM AG1 were added to the imaging solution and introduced into the chamber. The images of MTs were observed under total internal reflection fluorescence microscopy (IX81, Olympus) with a × 100 objective lens (UPlanSApo, numerical aperture (NA) 1.40, Olympus) and recorded at the frame rate of two frames per second using iXon3 EM CCD camera (Andor). Each MT image was rotated and translated to align the GMPCPP seed in the rhodamine channel by using custom scripts on the Fiji distribution of Image J (ref. 37). Then, the kymograph was generated to measure the dynamics of MT growth.

### *In vitro* MT polymerization assay

For glutamate-stimulated MT polymerization, 1 mg ml^−1^ porcine brain α/β-tubulin was mixed with glutamate (final concentration 1 M) and GTP (final concentration 1 mM) in RB buffer on ice. For paclitaxel-stimulated MT polymerization, 1 mg ml^−1^ porcine brain α/β-tubulin was mixed with paclitaxel (final concentration 10 μM) and GTP (final concentration 1 mM) in RB buffer on ice. For recombinant Tau protein-stimulated MT polymerization, 1 mg ml^−1^ α/β-tubulin purified from porcine brain and Tau protein were mixed with GTP (final concentration 1 mM) in RB buffer on ice. For γ-TuSC-stimulated MT polymerization, γ-TuSC was mixed with GTP (final concentration 1 mM). One of the compounds and α/β-tubulin were then added. Samples were transferred into cuvettes. Tubulin polymerization was monitored using the absorbance at 350 nm at 37 °C using a thermostatic spectrophotometer (Beckman Coulter).

### γ-TuSC capping to paclitaxel stabilized MT analysis

γ-TuSC was diluted in RB buffer containing 10 μM paclitaxel to reach a final protein concentration of 0.5 μM and incubated with 50 μM of gatastatin for 30 min at 25 °C. Polymerized MTs were prepared by incubation of ∼5 mg ml^−1^ α/β-tubulin for 30 min at 37 °C with 1 mM GTP; then paclitaxel was added (final 10 μM). GTP was removed from paclitaxel-stabilized MTs with ultracentrifugation at 240,000*g* for 10 min at 25 °C. Paclitaxel-stabilized MTs were added to γ-TuSC (final ∼1.2 mg ml^−1^). Next, γ-TuSC was incubated with paclitaxel-stabilized MTs for 30 min; MTs and MT-bound γ-TuSC were collected with ultracentrifugation at 240,000*g* for 10 min at 25 °C and finally placed on poly-L-lysine-coated coverslips. After incubation for 15 min, coverslips were immediately fixed with cold MeOH (−20 °C). Coverslips were blocked with PBS containing 0.5% bovine serum albumin and were incubated with anti-α-tubulin (1:200 dilution, MBL, Cat# PM054) and anti-penta His (1:200 dilution, Qiagen, Cat# 34660) antibodies. After staining with Alexa^488^-conjugated anti-rabbit IgG (1:2000 dilution, Invitrogen) and Alexa^568^-conjugated anti-mouse IgG (1:2000 dilution, Invitrogen), the coverslips were washed four times with PBS and mounted with PBS. γ-TuSC bound to MTs was observed under a LAS AF 6000 fluorescence microscope (Leica Microsystems, Wetzlar, Germany).

### *Xenopus* egg extract analysis

Cytostatic factor (CSF)-arrested M phase *Xenopus* egg extracts (CSF extracts) were prepared^38^. Briefly, unfertilized eggs were crushed by 10,700*g* centrifugation for 20 min. M phase extracts were mixed with 0.5 μM Cy3-labelled α/β-tubulin and various concentrations of compounds. After incubation for 30 min on ice, 15 μM RanQ69L, 200 sperm nuclei per μl or 5% DMSO were added and incubated for 30 min on ice. After incubation for 20 min at 20 °C (for the samples with sperm nuclei, the incubation time was 30 min), 1.5 μl sample was fixed with 1.5 μl fix solution (0.3 volume of 37% formaldehyde, 0.6 volume of 80% glycerol and 0.1 vol of 10 × MMR solution (50 mM HEPES, pH 7.8, 1 M NaCl, 20 mM KCl, 10 mM MgCl_2_, 20 mM CaCl_2_ and 1 mM EDTA)), followed by the squash fix.

For γ-tubulin depletion, freshly prepared egg extracts were incubated for 30 min with γ-tubulin antibodies coupled to Dynabeads Protein A (Thermo Fisher Scientific) three times^39^,^40^. The depletion efficiency was verified using western blot analysis.

Images were acquired using a microscope (Axiovert 200 M; Carl Zeiss Inc.) equipped with a plan-Apochromat × 63 NA 1.3 oil objective lens (Carl Zeiss Inc.), a Cy3 emission filter and a Cascade:1 K (Photometrics Inc.) camera, and the AxioVision software (Carl Zeiss Inc.). The aster size and fluorescence intensity were quantified using a macro written in MATLAB^41^.

### MT nucleation assay

HeLa and U2OS cells were cultured in DMEM+GlutaMAX (Gibco) supplemented with 10% fetal bovine serum (FBS), 100 U ml^−1^ penicillin, 100 μg ml^−1^ streptomycin and 1 mM sodium pyruvate in a humidified atmosphere containing 5% CO_2_. RPE-1 cells were cultured in DMEM/F-12 (Gibco) supplemented with 10% FBS, 2 mM L-glutamine, 100 U ml^−1^ penicillin, 100 μg ml^−1^ streptomycin, 1 mM sodium pyruvate containing 10% FBS in a humidified atmosphere containing 5% CO_2_.

To depolymerize MTs, interphase cells were treated with 10 μM of nocodazole for 3 h, washed with cold medium and incubated on ice for 1 h. Mitotic cells were treated with 10 μM of STLC for 6 h, washed with cold medium and incubated on ice for 1 h. Subsequently, both cells were incubated in medium containing 1% DMSO or 30 μM of gatastatin for 10 min on ice. Cells were then put in warmed medium containing either 1% DMSO or 30 μM of gatastatin and were incubated at 37 °C (interphase cells) or 20 °C (mitotic cells) for 1 min. The cells were immediately fixed with cold MeOH (−20 °C).

After blocking with PBS containing 0.5% bovine serum albumin, cells were incubated with anti-EB1 antibody (1:500 dilution, Abcam, Cat# ab53358), anti-α-tubulin antibody (1:500 dilution, Sigma, Cat# T9026) and anti-pericentrin antibody (1:2,000 dilution, Abcam, Cat# ab4448). After staining with Alexa^555^-conjugated anti-rat IgG (1:500 dilution, Molecular Probes), Alexa^647^-conjugated anti-mouse IgG (1:500 dilution, Molecular Probes), Alexa^488^-conjugated anti-rabbit IgG (1:500 dilution, Molecular Probes) and 5 μg ml^−1^ Hoechst 33342, cells were washed four times with PBS and mounted with ProLong Gold Antifade Reagent (Life Technologies). MT nucleation was observed under a DeltaVision Olympus IX71 microscope (Applied Precision) equipped with CoolSNAP HQ camera (Photometrics).

### EB3-EGFP signal tracking analysis

A HeLa ‘Kyoto' cell line stably expressing EB3-EGFP (a kind gift from Dr J. Ellenberg) was maintained in DMEM+GlutaMAX (Gibco) supplemented with 10% FBS, 2 mM L-glutamine, 100 U ml^−1^ penicillin, 100 μg ml^−1^ streptomycin, 1 mM sodium pyruvate and 0.5 mg ml^−1^ G418. Cells were seeded into eight-well Lab-Tek chambers (Nunc, Rochester, NY) for 24-h prior imaging. For imaging, the medium was exchanged to Live Cell Imaging Solution (Molecular Probes Life Technologies) supplemented as described above and containing various concentrations of AG1 or gatastatin. Interphase cells were incubated with drugs for 15 min and mitotic cells were treated as described in Fig. 4c. Then, EB3 tracks were observed under a PerkinElmer UltraVIEW ERS Spinning Disk microscope (Software: UltraVIEW ERS Imaging Suite) equipped with a Plan-APOCHROMAT × 100/1.4 Oil DIC objective, a Confocal Scanner Unit (Yokogawa CSU 22), a Hamamatsu EM CCD camera (C9100-50) and a 488-nm Argon laser at 37 °C, and time-lapse sequences were performed for 60 s with a 400-ms interval. Automatic analysis of EB3-EGFP signal tracks was performed with ImageJ (http://imagej.nih.gov/ij/) and Matlab (Mathworks) as described^16^.

### Analysis of interphase MT network and spindle structure

HeLa cells were cultured in DMEM containing 10% FBS in a humidified atmosphere containing 5% CO_2_. After treatment with each compound for 2.5 h (interphase MT network) or 5 h (mitotic spindle), the cells were immediately fixed with cold MeOH (−20 °C). After blocking with PBS containing 0.5% bovine serum albumin, cells were incubated with anti-α-tubulin antibodies (1:500 dilution, Santa Cruz, Cat# sc-32293) to observe interphase cells, or with anti-BubR1 (1:250 dilution, BD Bioscience, Cat# 612502) and anti-α-tubulin (1:250 dilution, MBL, Cat# PM054) antibodies to observe mitotic spindle. After staining with Alexa^488^-conjugated anti-mouse IgG (1:2,000 dilution, Invitrogen, Cat#A11001) and Alexa568-conjugated anti-rabbit IgG (1:2000 dilution, Invitrogen, Cat#A11011), cells were washed four times with PBS and mounted with PBS containing 0.1 μg ml^−1^ DAPI. MTs and BubR1 signals were observed under a Leica LAS AF 6000 fluorescence microscope (Leica Microsystems) or an LSM 700 laser-scanning confocal microscope (Zeiss).

### Measurement of spindle length of gatastatin-treated cells

Exponentially growing HeLa cells were treated with 30 μM of gatastatin for 9 h. Cells were fixed with cold MeOH (−20 °C). Immunostaining was performed as described above using anti-α-tubulin (1:250 dilution, Santa Cruz, Cat# sc-32293) and anti-pericentrin (1:250 dilution, Abcam, Cat# ab4448) antibodies. After staining with Alexa^488^-conjugated anti-mouse IgG (1:2,000 dilution, Invitrogen, Cat#A11001) and Alexa^568^-conjugated anti-rabbit IgG (1:2,000 dilution, Invitrogen, Cat#A11011), cells were washed four times with PBS and mounted with PBS containing 0.1 μg ml^−1^ DAPI. Spindle structure was observed under a Leica LAS AF 6000 fluorescence microscope (Leica Microsystems). Images of 41 sections at 0.251-μm intervals were collected. The position of the two pericentrin signals of mitotic cells was analysed using the ImageJ software, and then their distance was calculated.

### Analysis of the transition from S phase to mitotic entry

Cells expressing EGFP-α-tubulin and mCherry-H2B were prepared as described previously^42^. Cells were arrested at the S phase with 2-mM thymidine treatment for 15 h. To determine the time required for mitotic entry from S phase release, thymidine was removed by washing cells with PBS three times. After treatment with 30 μM gatastatin for 9 h, time-lapse analysis was performed using DeltaVision Olympus IX71 microscope (Applied Precision) equipped with CoolSNAP HQ camera (Photometrics). Images were taken every 6 min with 12 z-stack (1 μm per stack). During live cell imaging, cells were maintained at 37 °C and were supplied with 5% CO_2_. The timing of the mitotic entry was defined by the chromosome condensation after nuclear envelope breakdown.

### Bipolar spindle establishment and spindle length

To analyse bipolar spindle establishment from monopolar spindles, HeLa cells were arrested at prometaphase with 10 μM of STLC for 15 h. STLC was removed by washing cells with the medium three times. After treatment with 1% DMSO or 30 μM of gatastatin for 2 h, cells were immediately fixed with cold MeOH (−20 °C).

For analysis of the length of the bipolar spindle in MG132-treated cells, HeLa cells were arrested at prometaphase with 10 μM of STLC for 15 h, followed by STLC removal by washing the cells with the medium three times to remove STLC. After treatment with 5 μM of MG132 for 2 h, 1% DMSO or 30 μM of gatastatin was added. Cells were incubated for 2 h (combined treatment with MG132 and gatastatin) and then immediately fixed with cold MeOH (−20 °C).

Immunostaining was performed as described above using anti-α-tubulin (1:250 dilution, Santa Cruz, Cat# sc-32293) and anti-pericentrin (1:250 dilution, Abcam, Cat# ab4448) antibodies. Spindle structure was observed under a Leica LAS AF 6000 fluorescence microscope (Leica Microsystems). The measurement of spindle length was performed as described above.

### Analysis of gatastatin-treated spindle using 3D-SIM

To observe the effect of gatastatin on MG132-treated metaphase-like spindle using 3D-SIM (three-dimensional structural illumination microscopy), HeLa cells were arrested at metaphase by sequential treatment with STLC and MG132. After treatment with 1% DMSO or 30 μM of gatastatin for 2 h (combination treatment with MG132 and gatastatin), cells were fixed with cold MeOH (−20 °C). Cells were blocked with PBS containing 0.5% bovine serum albumin and then were incubated with anti-α-tubulin (1:500 dilution, Sigma-Aldrich Cat# T9026) and γ-tubulin (1:500 dilution, antibody against C-EYHAATRPDYISWGTQDK peptide of γ-tubulin^43^) antibodies. Secondary antibodies were Alexa^488^-conjugated anti-mouse IgG (1:500 dilution, Molecular Probes) and Alexa555-conjugated anti-rabbit IgG (1:500 dilution, Molecular Probes). DNA was stained with 0.5 μg ml^−1^ Hoechst 33342 for 30 min. Finally, cells were washed four times with PBS and mounted in Prolong Gold Antifade (Molecular Probes, #P36930). Spindle structure was observed under a 3D-SIM-Nikon Ti inverted microscope.

### Analysis of the anaphase spindle elongation and cytokinesis

HeLa cells expressing mCherry-H2B and EGFP-α-tubulin, HeLa ‘Kyoto' cell line stably expressing CEP55-LAPtag (a kind gift from Dr A.A. Hyman) and HeLa cells expressing RFP-H2B and GFP-LifeAct (a kind gift from Dr J. Ellenberg) were cultured in DMEM+GlutaMAX (Gibco) supplemented with 10% FBS, 100 U ml^−1^ penicillin, 100 μg ml^−1^ streptomycin and 1 mM sodium pyruvate in a humidified atmosphere containing 5% CO_2_. To observe the transition from metaphase to anaphase, telophase and cytokinesis, cells were arrested at metaphase by sequential treatment of STLC and MG132. After MG132 removal by washing of cells with medium three times, the medium was changed to Live Cell Imaging Solution (Molecular Probes Life Technologies) supplemented as described above containing 1 μM of MPS1 inhibitor reversine and either DMSO or 30 μM of gatastatin. Time-lapse observation was performed at 37 °C using the DeltaVision Olympus IX71 microscope (Applied Precision) equipped with CoolSNAP HQ camera (Photometrics). Images were taken every 3 min with 12 z-stacks (1 μm per stack) for 3 h. The anaphase duration (between chromosome separation and start of midbody formation) was calculated.

## Additional information

**How to cite this article:** Chinen, T. *et al.* The γ-tubulin-specific inhibitor gatastatin reveals temporal requirements of microtubule nucleation during the cell cycle. *Nat. Commun.* 6:8722 doi: 10.1038/ncomms9722 (2015).

## Supplementary Material

Supplementary Information Supplementary Figures 1-15, Supplementary Notes 1-2 and Supplementary References

## Figures and Tables

**Table 1:** Drug-binding analysis based on tryptophan fluorescence spectrum changes.

	***K***_**d**_ (μM)*		**Ratio of** ***K***_**d**_^**[α/β-tubulin]**^**/*****K***_**d**_^**[γ-tubulin]**^
	**α/β-Tubulin**	**γ-Tubulin**	
Colchicine	17.5±2.7	196.4±47.9	0.09
Nocodazole	1.8±0.6	54.9±19.0	0.03
Plinabulin	0.5±0.1	83.3±18.5	0.01
KPU-406	26.3±2.0	46.1±27.6	0.57
AG1	51.9±36.4	85.3±22.8	0.61
Gatastatin	42.5±36.7	3.6±1.3	11.81

^*^The *K*_d_ value of each drug was calculated from tryptophan fluorescence decrease (ΔFL) from three independent experiments using the GraphPad Prizm software. Values are the mean±s.d.

**Table 2:** The effect of AG1 and gatastatin on GTP-binding activity of γ-tubulin *in vitro*.

**Compound**	**Concentration (μM)**	**GTP binding (control %)**
AG1	60	93.7±11.7
Gatastatin	60	6.7±3.7

GTP-binding inhibition (control %) by compounds was calculated as described in Methods section. Values are the mean±s.d.

**Table 3:** Effects of AG1 and gatastatin on the MT dynamics in interphase cells.

**Compound**	**Conc. (μM)**	**Velocity (μm min**^**−1**^**)**	**Track length (μm)**	**Track lifetime (s)**	Track numbers*
DMSO		12.70±1.65	1.08±0.14	3.32±0.55	87±21
AG1	1	9.59±1.01**	0.73±0.09**	3.30±0.57	106±30*
	3	7.33±1.23**	0.43±0.07**	2.20±0.67**	110±42*
Gatastatin	3	12.49±1.67	1.03±0.16	3.45±0.71	94±27
	10	10.03±0.99**	0.84±0.10**	3.67±0.62	83±30
	30	8.43±0.81**	0.71±0.08**	3.84±0.50*	89±32

Conc., concentration; DMSO, dimethylsulphoxide; MT, microtubule.

EB3-EGFP tracks from cells (Fig. 3b) were analysed as described in Methods section. Values are the mean±s.d. from 10,543 to 20,978 tracks from 20 to 35 cells *P<0.05, **P<0.0001. Two-tailed, unpaired Student's *t*-test was used to obtain *P* value by comparison with DMSO control.

^*^Track numbers from a 144-μm^2^ area inside the cell were calculated as described in Methods section. Values are the mean±s.d. from 20 to 35 cells.

**Table 4:** Effects of gatastatin on the MT dynamics in mitotic cells.

**Compound**	**Conc. (μM)**	**Velocity (μm min**^**−1**^**)**	**Track length (μm)**	**Track lifetime (s)**	Track numbers*
DMSO		13.35±0.92	0.67±0.09	1.64±0.42	44.67±7.07
Gatastatin	30	11.21±1.68**	0.53±0.09**	1.76±0.66	37.27±8.12**

Conc., concentration; DMSO, dimethylsulphoxide; MT, microtubule.

EB3-EGFP tracks from mitotic cells of Fig. 4e condition were analysed as described in Methods section. Values are the mean±s.d. from 9,146 to 11,403 tracks from 15 to 18 cells from three independent experiments **P<0.0001. Two-tailed, unpaired Student's *t*-test was used to obtain *P* value by comparison with DMSO control.

^*^Track numbers from a 25-μm^2^ area in the spindle were calculated as described in Methods section. Values are the mean±s.d. from 15 to 18 cells.
