# Supplementary material for: The γ-tubulin-specific inhibitor gatastatin reveals temporal requirements of microtubule nucleation during the cell cycle
Source: Nat Commun. 2015 Oct 27;6:8722. doi: 10.1038/ncomms9722 (PMC4640066; doi:10.1038/ncomms9722)
Supplement: Supplementary Information — Supplementary Figures 1-15, Supplementary Notes 1-2 and Supplementary References [file ncomms9722-s1.pdf]

Supplementary Figures

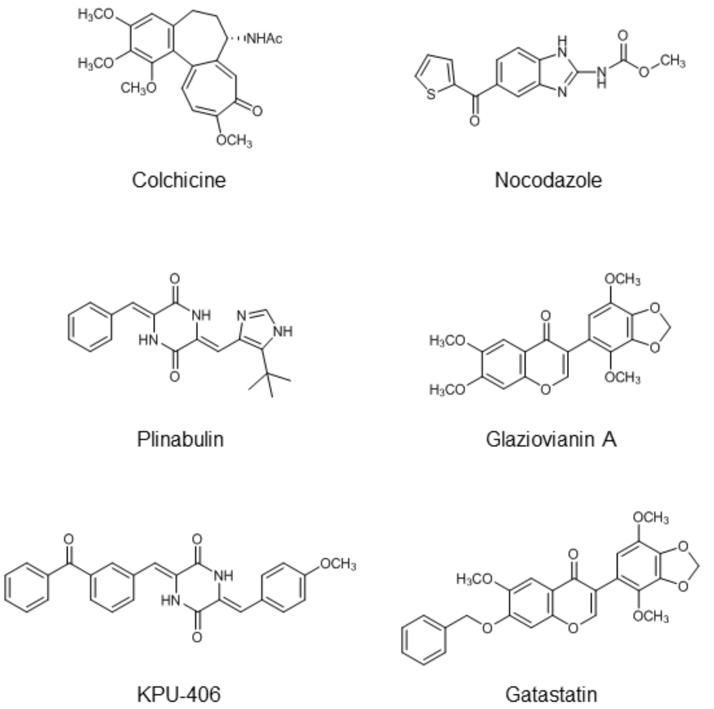

Supplementary Figure 1. Chemical structures of compounds used in this study
Shown are the structures of colchicine, nocodazole, plinabulin, KPU-406, glaziovianin A (AG1), and gatastatin (O^7-demethylbenzyl AG1).

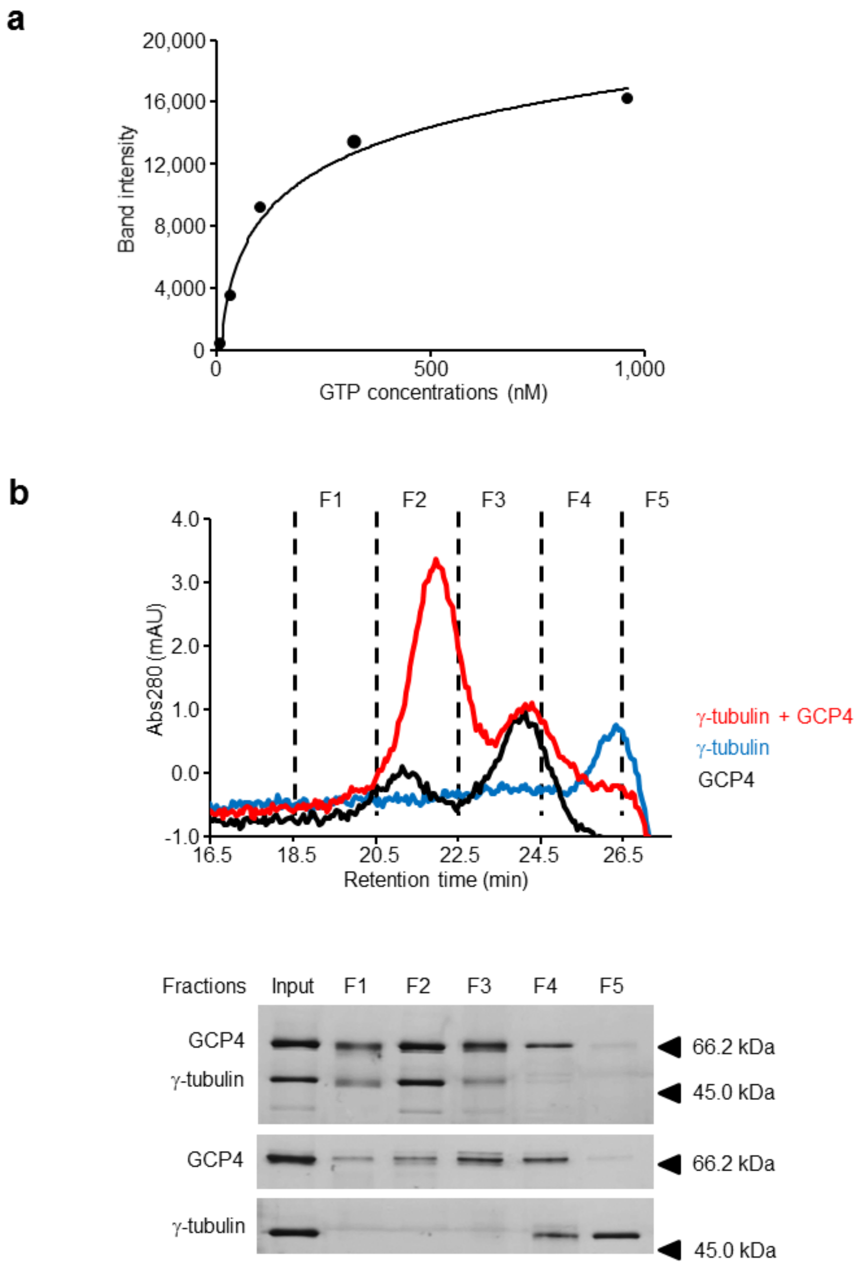

Supplementary Figure 2. Binding of GTP and GCP4 to recombinant human γ-tubulin

(**a**) GTP binding to γ-tubulin. 70 nM of γ-tubulin was mixed with indicated concentrations of α-[^{32}P]-GTP, and cross-linked by UV irradiation. After separation of crosslinked γ-tubulin and unbound α-[^{32}P]-GTP, radioactivity was detected with a phosphorimager. Band intensities were quantified with ImageJ. The K_d (121.1 nM) was calculated using GraphPad Prizm software. (**b**) Purified human γ-tubulin and GCP4 interaction was detected by gel filtration and SDS-PAGE. Equal volumes of γ-tubulin (1.5 µM) and GCP4 (4 µM), γ-tubulin (1.5 µM) and buffer, or GCP4 (4 µM) and buffer were mixed and incubated for 30 min at 20ºC. Proteins were separated by size exclusion column Superdex 200 10/300 GL pre-equilibrated with gel filtration buffer (50 mM MES pH6.6, 500 mM KCl, 5 mM $MgSO_4$, 1 mM EGTA, 1 mM DTT, 10 µM GTP). γ-Tubulin alone and GCP4 alone were eluted at retention times of 27 min and 24 min, respectively. The mixture of γ-tubulin and GCP4 had a retention time of 21 min. GCP4 and γ-tubulin in these fractions were confirmed by SDS-PAGE and silver staining.

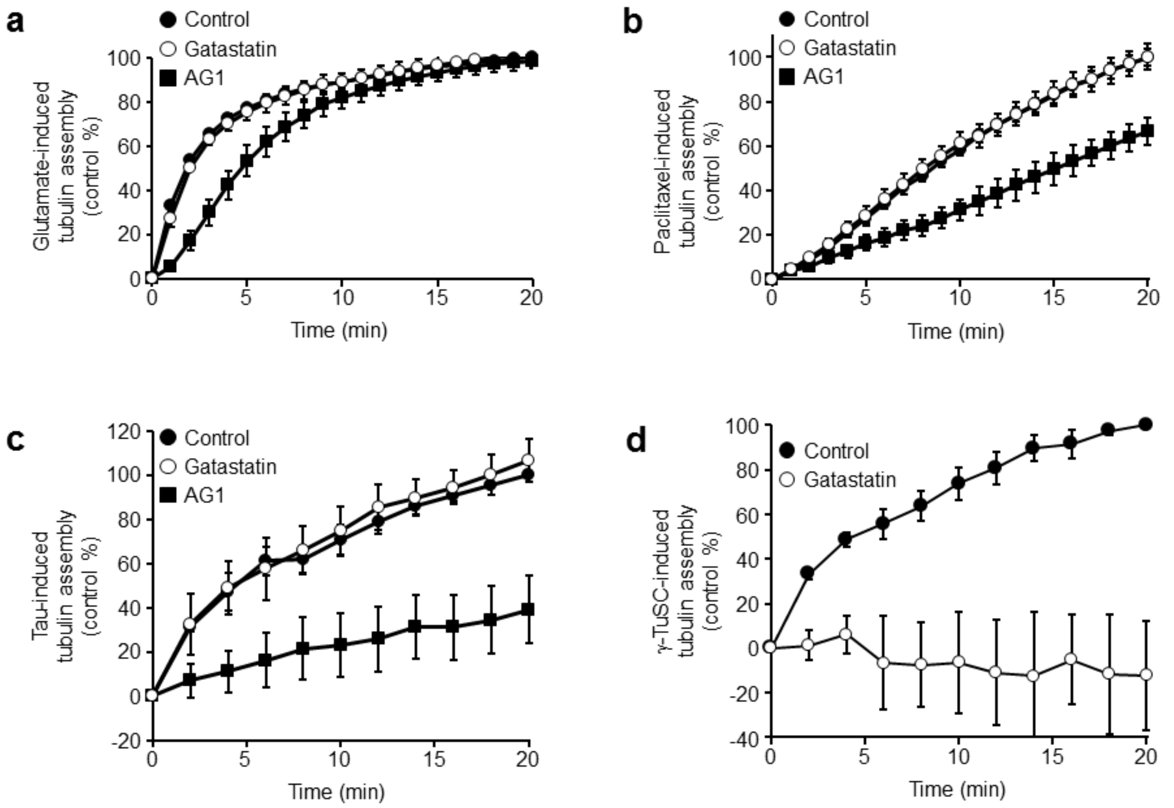

Supplementary Figure 3. The effects of gatastatin on MT polymerization *in vitro*

(**a-c**) Gatastatin did not affect γ-tubulin-independent MT polymerization. 1 mg/ml purified porcine brain α/β-tubulin and glutamate (**a**), paclitaxel (**b**) or recombinant Tau protein (**c**) were mixed with each compound. Tubulin polymerization was determined by measuring OD at 350 nm. In (**a**), closed and open circles are the control and 100 μM gatastatin, respectively. Closed squares are 10 μM AG1. In (**b**), closed and open circles are the control and 30 μM of gatastatin, respectively. The closed squares represent 30 μM of AG1. In (**c**), closed and open circles are the control and gatastatin, respectively. The closed squares represent 30 μM of AG1. (**d**) The effect of gatastatin on the γ-tubulin-dependent MT polymerization. 1 mg/ml porcine brain α/β-tubulin, γ-TuSC were mixed with 30 μM gatastatin. Closed and open circles are the control and 30 μM of gatastatin, respectively. (**a-d**) Tubulin polymerization was determined by measuring the OD at 350 nm. Error bars represent s. e. m from three independent experiments.

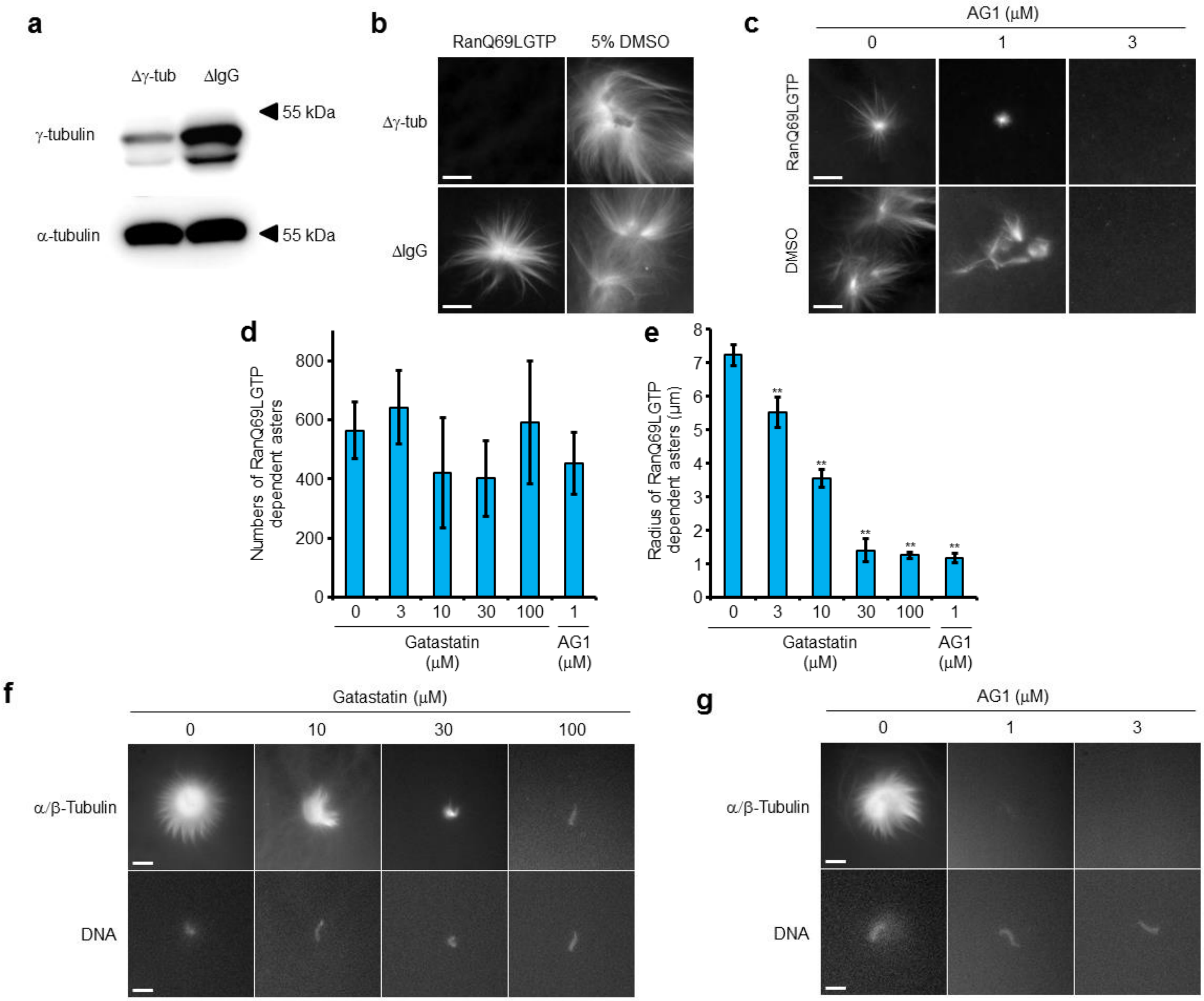

Supplementary Figure 4. Gatastatin inhibits γ-tubulin-depended RanQ69L- and sperm nuclei-induced aster formation but not γ-tubulin-independent DMSO-induced aster formation

(**a**) The efficiency of γ-tubulin depletion in *Xenopus* extracts was confirmed by immunoblotting. IgG was used as a control for depletion. The depletion efficiency was calculated with ImageJ and was approx. 80%. (**b**) RanQ69L- but not DMSO-induced aster formation requires γ-tubulin. γ-Tubulin depleted (Δγ-tub) or control IgG incubated (ΔIgG) extracts were incubated with RanQ69L or 5% DMSO at 20°C for 20 min. Aster formation was observed with a fluorescence microscope. Scale bars, 5 μm. (**c**) The effects of AG1 on the RanQ69L- and DMSO-stimulated aster formation. Egg extracts with Cy3-tubulin and AG1 were incubated for 20 min at 20°C in the presence of RanQ69L or 5% DMSO. Aster formation was analyzed by fluorescence microscopy. Scale bar, 5 μm. (**d**) The effects of gatastatin and AG1 on the RanQ69L-dependent aster number. Asters (Fig. 1e and Supplementary Figure 4**c**) in 10 randomly selected fields were counted per slide with a 10× objective; each sample had four slides. Three independent experiments were performed. Error bars represent S.D. Two-tailed, paired Student's *t*-test was used to obtain *P* values. There are no significant differences between control (no drug) and drug treated samples ($P > 0.05$). (**e**) Quantification of the radius of RanQ69L-induced asters from Fig. 1e and Supplementary Figure 4**c.** To gauge the impact upon the radius of the asters, n = 20 asters were quantified per sample per experiment. The images were acquired with a 63x objective. The effect of gatastatin (**f**) and AG1 (**g**) on sperm nuclei induced aster formation. *Xenopus* extracts were incubated with sperm nuclei at 20°C for 30 min. Aster formation was observed with a 40x objective. Scale bars, 5 μm.

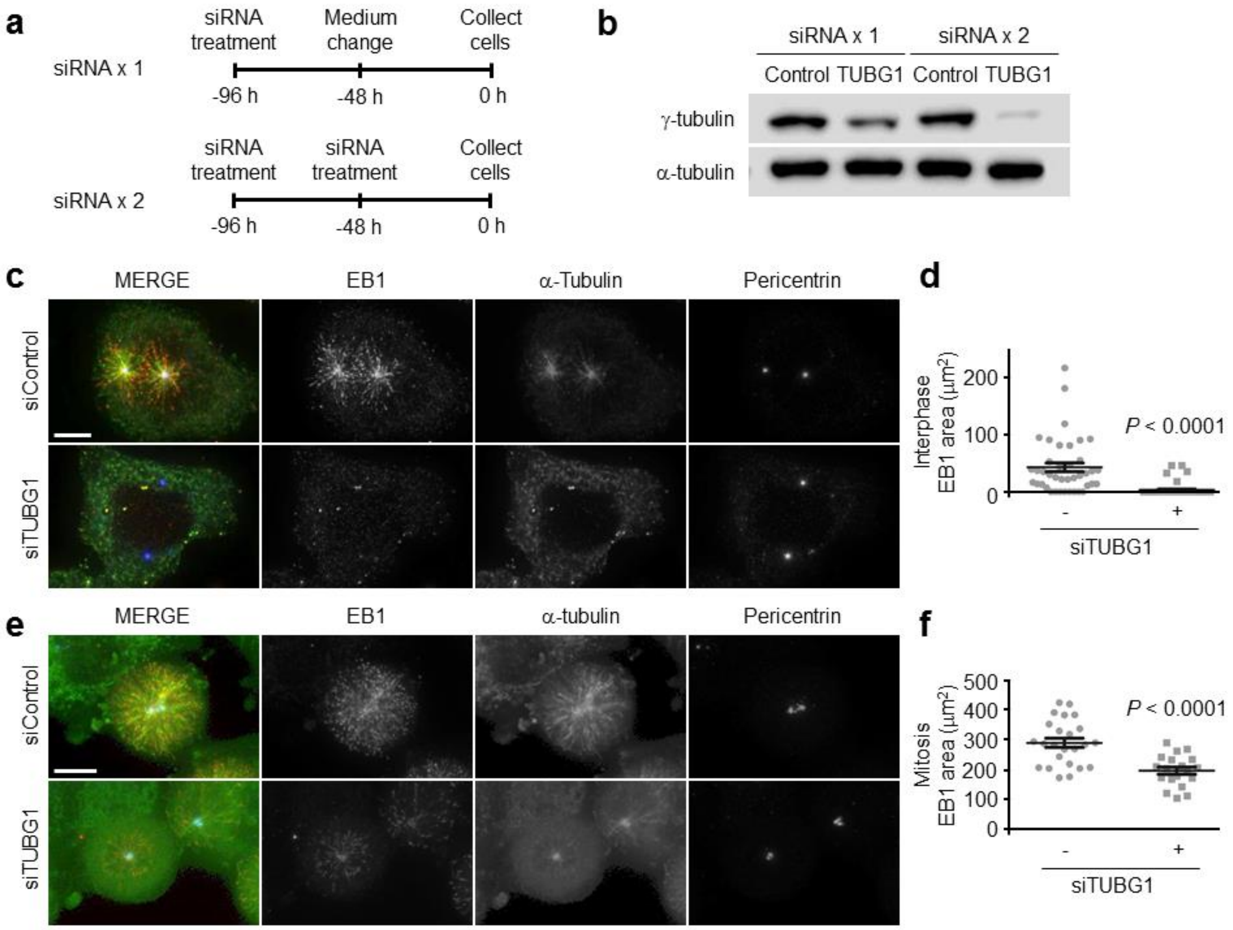

Supplementary Figure 5. Impact of γ-tubulin siRNA depletion on MT regrowth in HeLa cells
(**a**) Scheme of the siRNA treatment. (**b**) The efficiency of γ-tubulin depletion in HeLa cells was confirmed by immunoblotting. The efficiency of one round of γ-tubulin depletion with siRNA (final 40 nM, siTUBG1 Darmacon, SMARTpool, Cat# L-005160-00-0005) was only partial, but two repeated treatments drastically reduced the amount of γ-tubulin to > 90%. Two siRNA treatments were used to deplete γ-tubulin for following experiments. (**c**) The impact of γ-tubulin knockdown on MT nucleation by centrosomes in interphase. After treatment with nocodazole and incubation on ice, MT nucleation (1 min at 37°C) of γ-tubulin knock down cells was monitored. Red, green and blue in the merged picture represent EB1, α-tubulin and pericentrin, respectively. Scale bar, 10 μm. Three independent experiments were performed and one representative experiment is shown. (**d**) The area of EB1 around centrosomes in (**c**) was calculated using ImageJ software from 40 (control) or 52 (siTUBG1) centrosomes. Error bars represent s. e. m. Two-tailed, un-paired Student's *t*-test was used to obtain *P* values. (**e**) The impact of γ-tubulin knockdown on MT nucleation by centrosomes in mitosis. After treatment with STLC and incubation on ice, MT nucleation (1 min at 20°C) of γ-tubulin knock down cells was monitored upon shifting cells to room temperature. Red, green and blue in the merged picture represent EB1, α-tubulin and pericentrin, respectively. Scale bar, 10 μm. Three independent experiments were performed and one representative data set is shown. (**f**) The area of EB1 around centrosomes in (**e**) was calculated using ImageJ software from 24 (control) or 19 (siTUBG1) monopolar spindles. Error bars represent s. e. m. Two-tailed, un-paired Student's *t*-test was used to obtain *P* values.

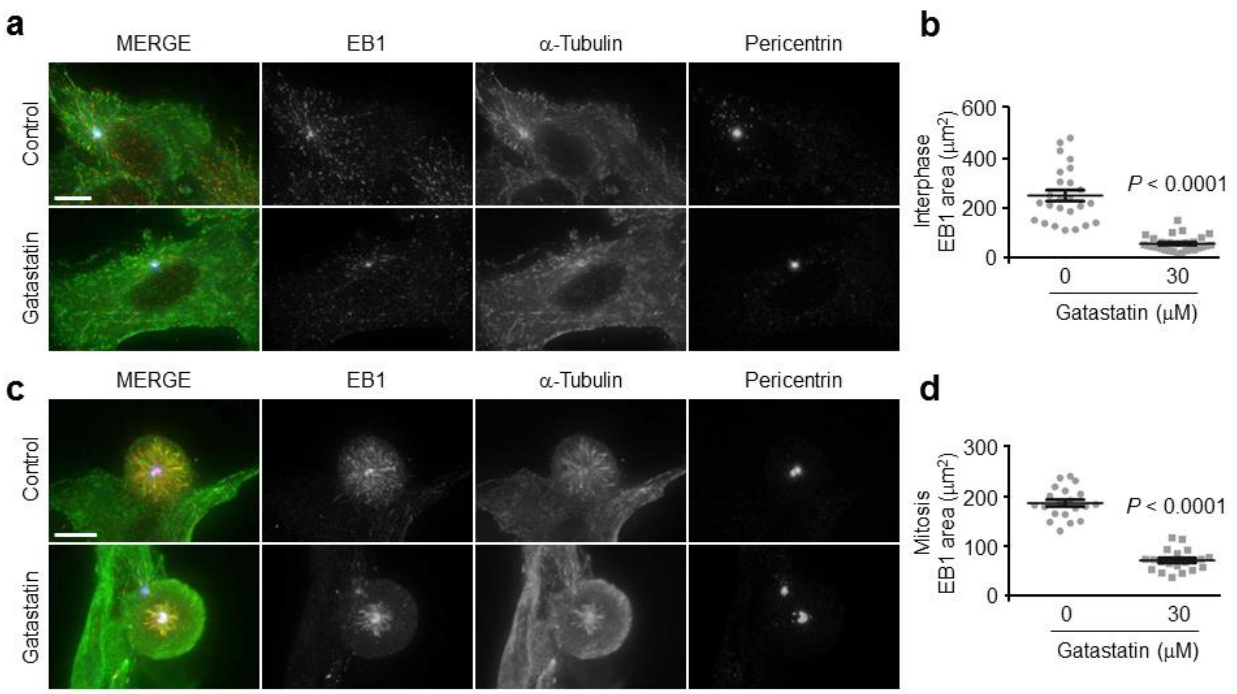

Supplementary Figure 6. Impact of gatastatin on MT regrowth in RPE1 cells

(**a**) The impact of gatastatin on MT nucleation by centrosomes in interphase. After nocodazole and ice-cold treatments, MT nucleation (nocodazole wash out, 1 min at 37°C) in the presence of 30 μM of gatastatin or the DMSO solvent control was monitored. Red, green and blue in the merged image represent EB1, α-tubulin and pericentrin, respectively. Scale bar, 10 μm. Three independent experiments were performed and one representative experiment is shown. (**b**) The area of EB1 around centrosomes in (**a**) was calculated using ImageJ software from 25 (control) or 29 (gatastatin) centrosomes. Error bars represent s. e. m. Two-tailed, un-paired Student's *t*-test was used to obtain *P* values. (**c**) The impact of gatastatin on MT nucleation by centrosomes in mitosis. After treatment with STLC and incubation on ice, spindle MT nucleation (1 min at 20°C) in the presence of 30 μM of gatastatin or the DMSO solvent control was monitored. Red, green and blue in the merged image represent EB1, α-tubulin and pericentrin, respectively. Scale bar, 10 μm. Three independent experiments were performed and one representative experiment is shown. (**d**) The area of EB1 around centrosomes in (**c**) was calculated using ImageJ software from 20 (control) or 20 (gatastatin) monopolar spindles. Error bars represent s. e. m. Two-tailed, un-paired Student's *t*-test was used to obtain *P* values.

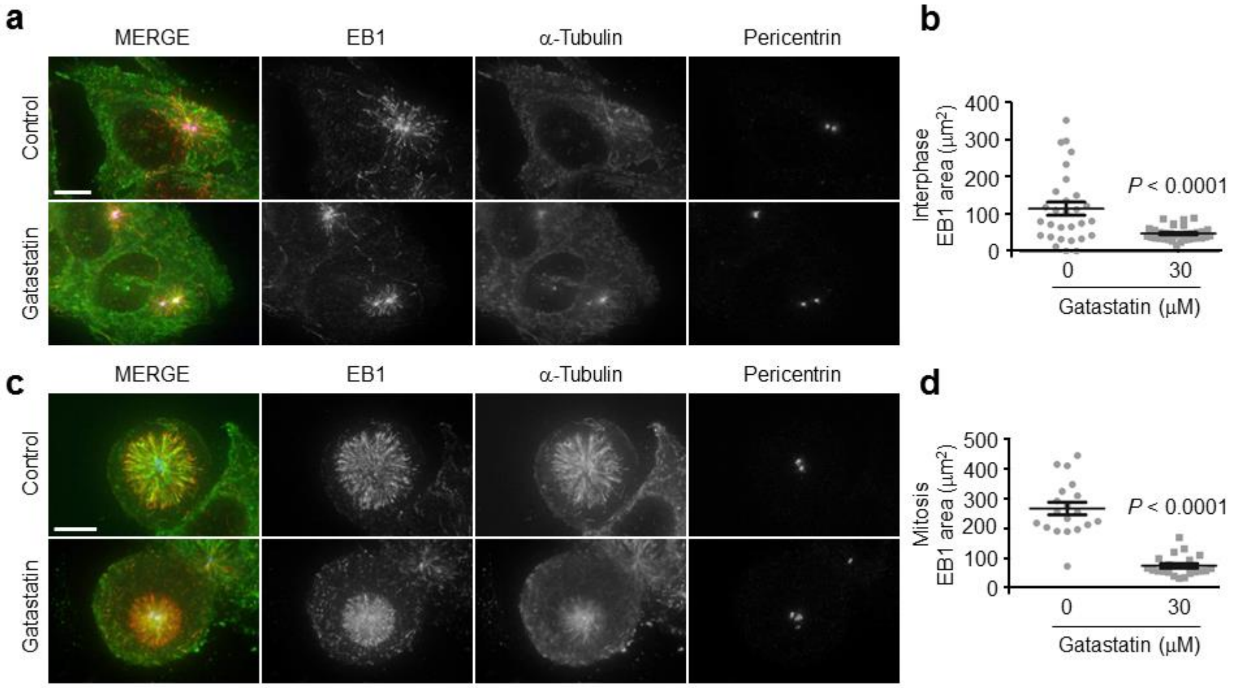

Supplementary Figure 7. Impact of gatastatin on MT regrowth in U2OS cells
(**a**) The impact of gatastatin on MT nucleation by centrosomes in interphase. After nocodazole and ice-cold treatments, MT nucleation (nocodazole wash out, 1 min at 37°C) in the presence of 30 μM of gatastatin or the DMSO solvent control was monitored. Red, green and blue in the merged image represent EB1, α-tubulin and pericentrin, respectively. Scale bar, 10 μm. Three independent experiments were performed and representative data set is shown. (**b**) The area of EB1 around centrosomes in (**a**) was calculated using ImageJ software from 29 (control) or 36 (gatastatin) centrosomes. Error bars represent s. e. m. Two-tailed, un-paired Student's *t*-test was used to obtain *P* values. (**c**) The impact of gatastatin on MT nucleation by centrosomes in mitosis. After treatment with STLC and incubation on ice, spindle MT nucleation (1 min at 20°C) in the presence of 30 μM of gatastatin or the DMSO solvent control was monitored. Red, green and blue in the merged image represent EB1, α-tubulin and pericentrin, respectively. Scale bar, 10 μm. Three independent experiments were performed and one representative experiment is shown. (**d**) The area of EB1 around centrosomes in (**c**) was calculated using ImageJ software from 19 (control) or 21 (gatastatin) monopolar spindles. Error bars represent s. e. m. Two-tailed, un-paired Student's *t*-test was used to obtain *P* values.

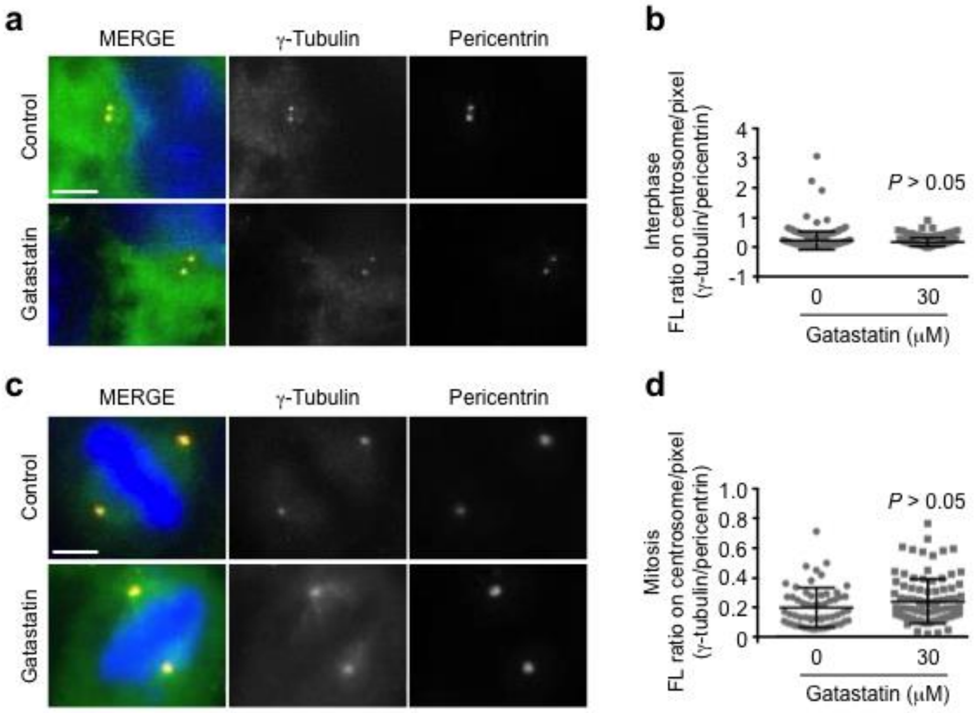

Supplementary Figure 8. Impact of gatastatin on γ-tubulin localization in HeLa cells

(**a-d**) γ-Tubulin localization on centrosome in gatastatin treated HeLa cells. (**a**) Gatatstatin-treated interphase cells (for 6 h) were analyzed. Red, green and blue in the merged image represent pericentrin, γ-tubulin and DNA, respectively. Scale bar, 5 μm. (**b**) The γ-tubulin signal around pericentrin in (**a**) was calculated using ImageJ software from 250 (control) and 166 (gatastatin) centrosomes. Error bars represent S.D. Two-tailed, un-paired Student's *t*-test was used to obtain *P* values. (**c**) Gatatstatin-treated mitotic cells (for 6 h) were analyzed. Red, green and blue in the merged image represent pericentrin, γ-tubulin and DNA, respectively. Scale bar, 5 μm. (**d**) The γ-tubulin signal on pericentrin in (**c**) was calculated using ImageJ software from 59 (control) and 98 (gatastatin) centrosomes. Error bars represent S.D. Two-tailed, un-paired Student's *t*-test was used to obtain *P* values. γ-Tubulin and pericentrin antibodies were purchased from Sigma (GTU-88, Cat# T6557) and Abcam (Cat# ab4448), respectively.

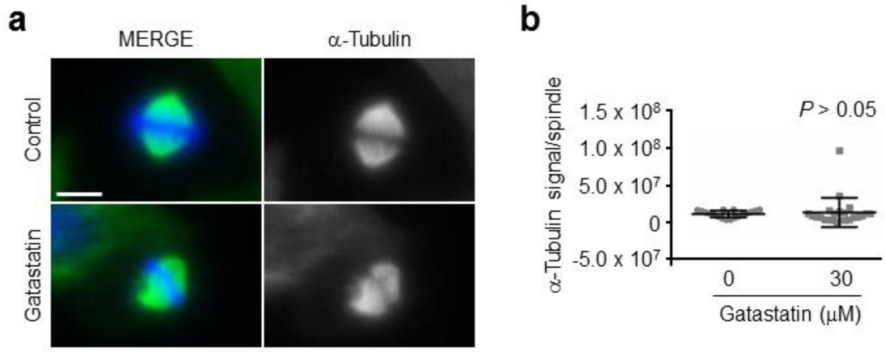

Supplementary Figure 9. Impact of gatastatin on α-tubulin density in mitotic spindles
(**a**) Tubulin density (antibody is from Sigma, DM1A, Cat# T9026) in gatastatin treated mitotic spindles (6 h, HeLa cells) was observed. Green and blue represent α-tubulin and DNA, respectively. Scale bar, 10 μm. Three independent experiments were performed and one representative data is shown. (**b**) The α-tubulin signal in the mitotic spindle in (**a**) was calculated using ImageJ software from 18 (control) or 23 (gatastatin) mitotic spindles. Error bars represent S.D. Two-tailed, un-paired Student's *t*-test was used to obtain *P* values.

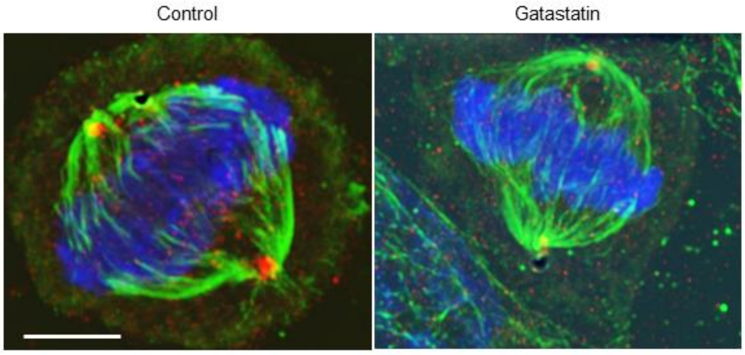

Supplementary Figure 10. Observation of the gatastatin treated spindle by SIM

Spindle structure of metaphase cells from experiment shown in Fig. 4c was analysed using SIM. Green, red and blue colors represent α-tubulin, γ-tubulin and DNA, respectively. Scale bar, 5 μm.

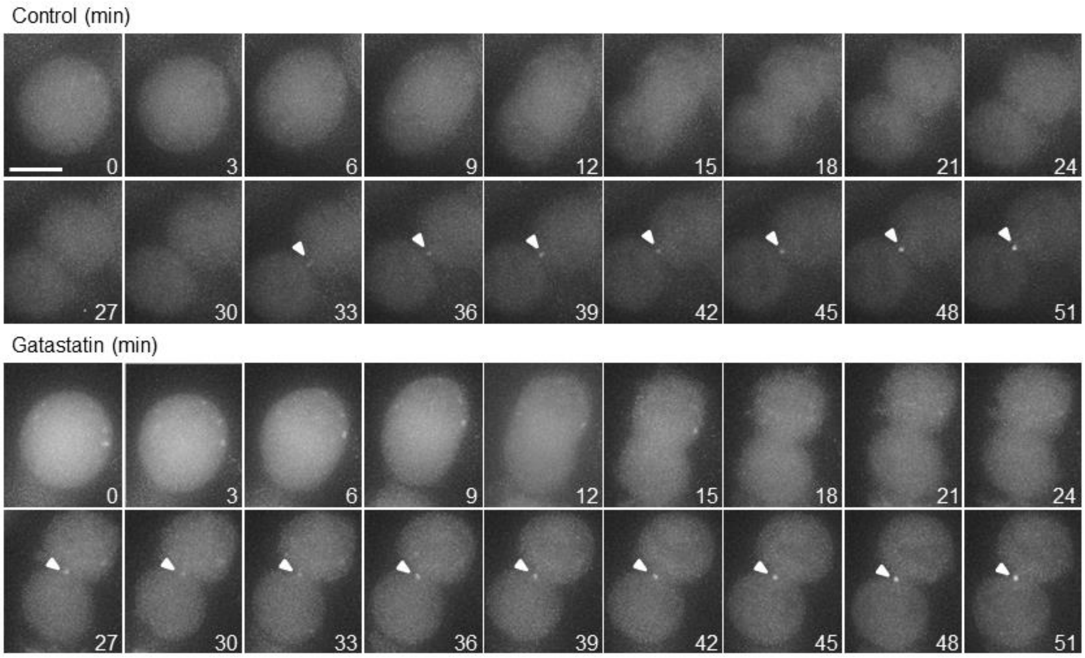

Supplementary Figure 11. Observation of the CEP55 localization in gatastatin treated cells

The experimental set up is described in Fig. 5a. CEP55-EGFP expressing HeLa cells were used. CEP55-EGFP localization in the midbody was observed with a 60x objective as described in the Method section. Arrowheads represents CEP55-EGFP localization in midbody. Scale bar, 5 µm.

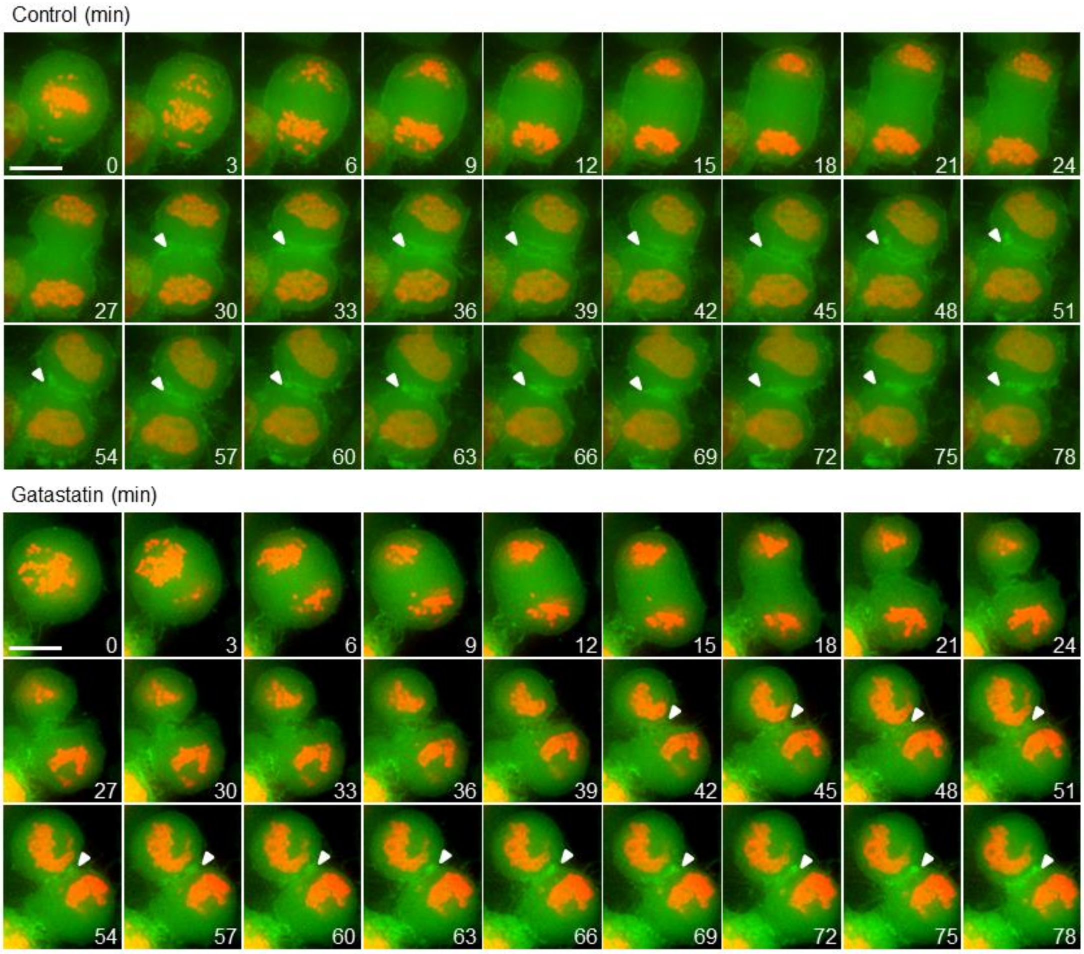

Supplementary Figure 12. Observation of the actin ring formation in gatastatin treated cells
The experimental set up is described in Fig. 5a. GFP-LifeAct and RFP-H2B expressing HeLa cells were used. Actin ring formation in midbody was observed with a 60x objective as described in the Method section. Arrowheads point towards the actin ring during cytokinesis. Note, it is difficult to compare timing of actin ring formation between control and gatastain treated cells because of the delay in anaphase spindle formation by gatastatin. Scale bar, 5 μm.

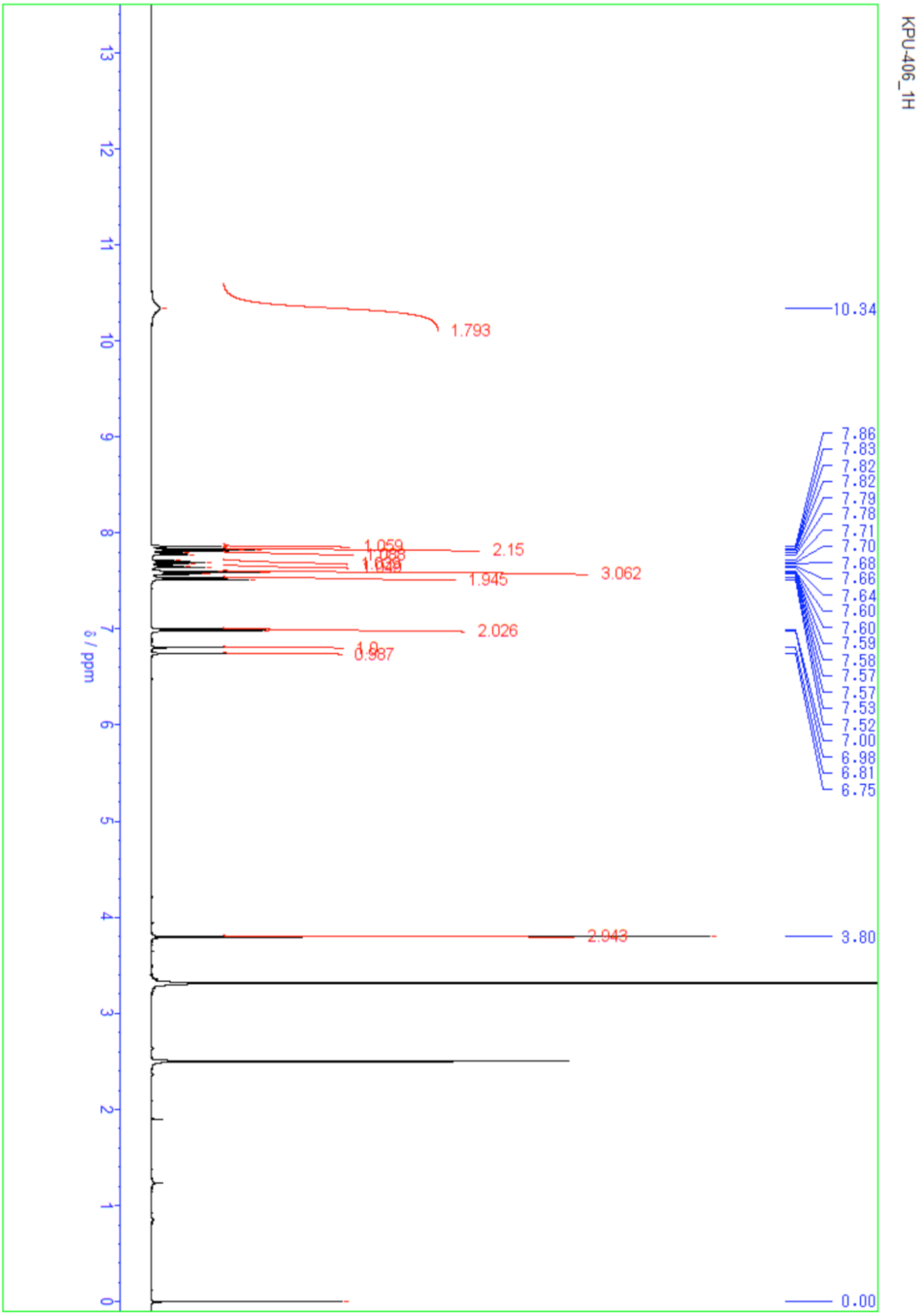

Supplementary Figure 13. ^{1}H NMR spectra of KPU-406.

^{1}H NMR spectra of KPU-406 (500 MHz, DMSO-d_6)

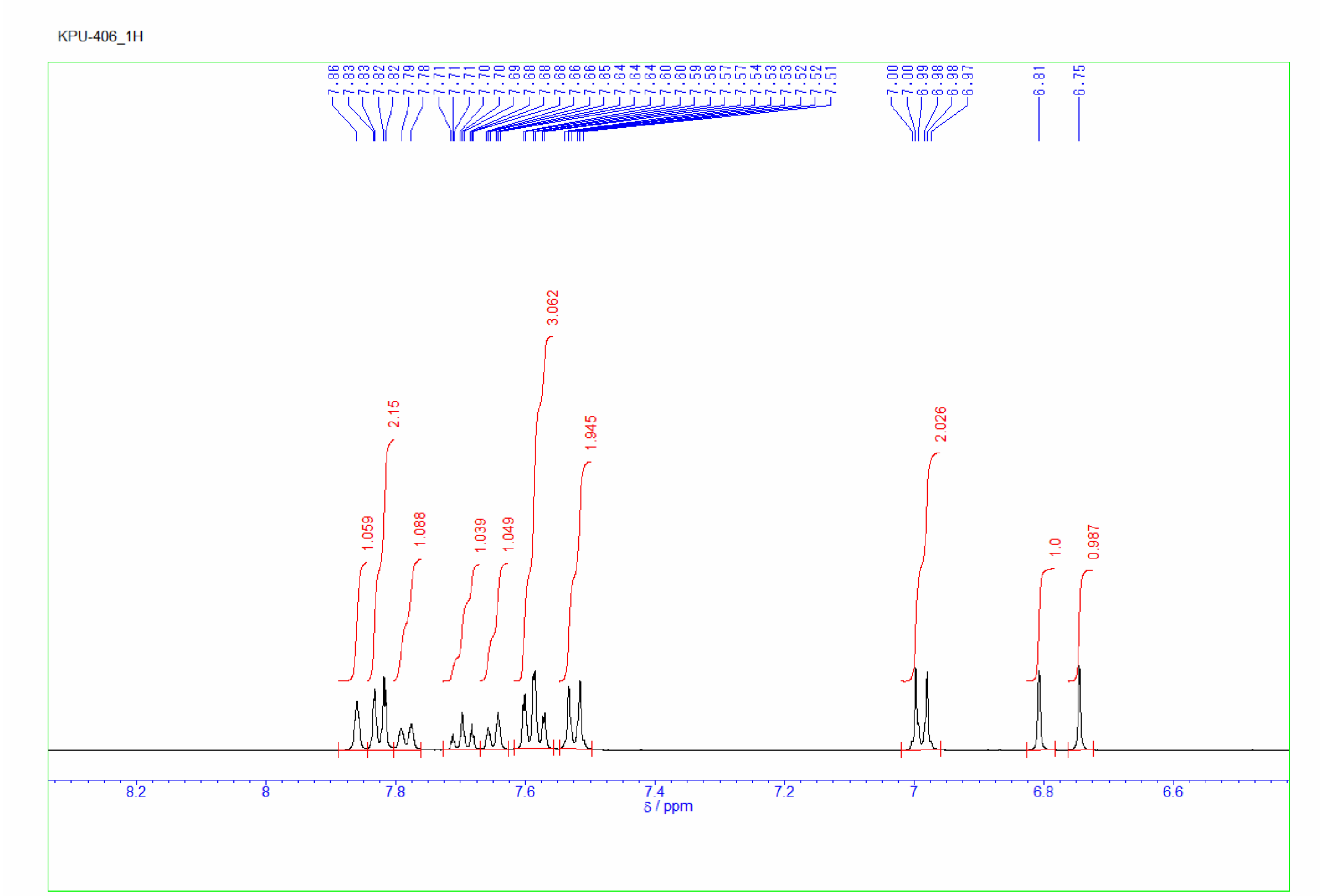

Supplementary Figure 14. ^{1}H NMR spectra of KPU-406 (enlarged).

^{1}H NMR spectra of KPU-406 (500 MHz, DMSO-d_6)

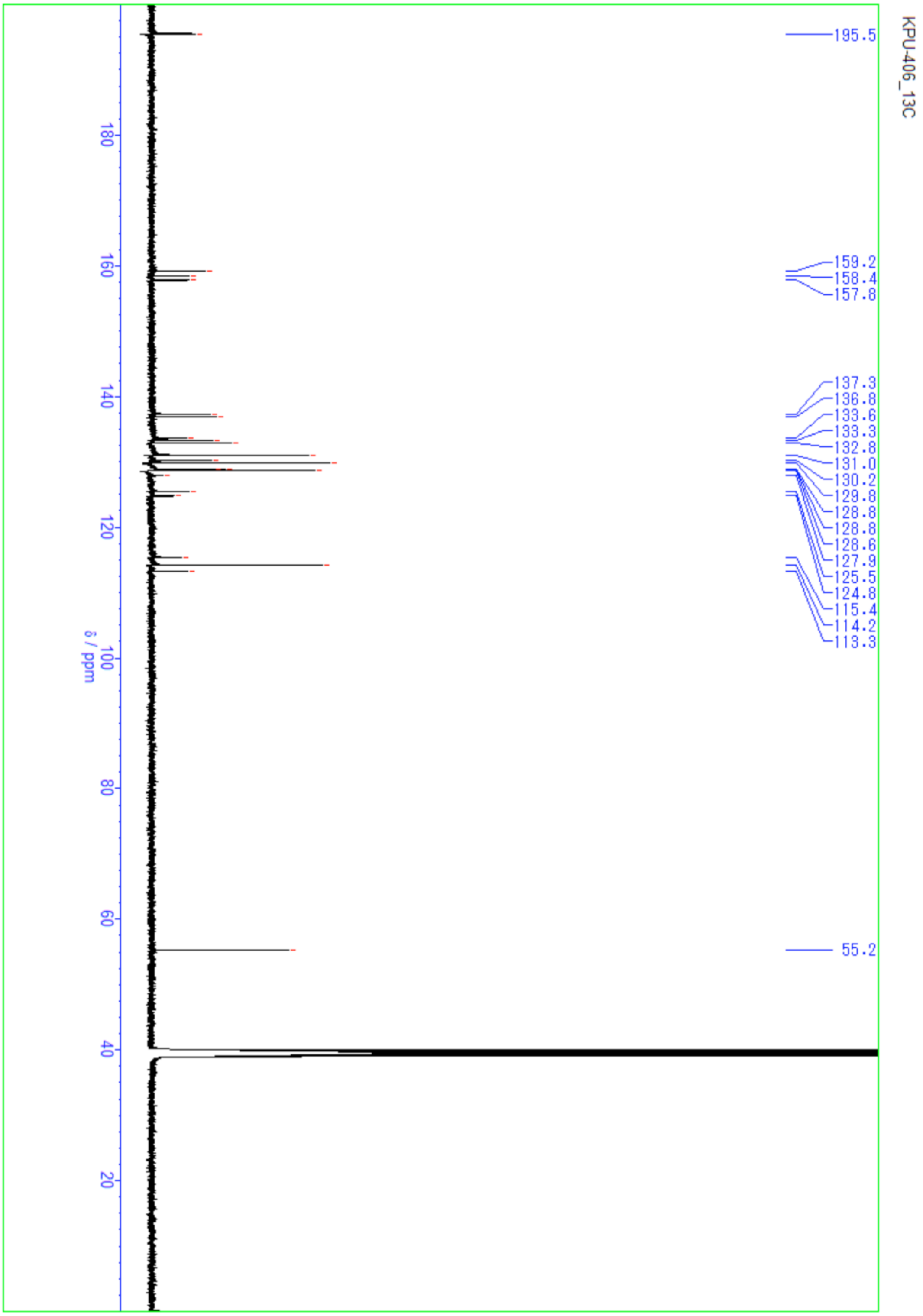

Supplementary Figure 15. ^{13}C NMR spectra of KPU-406.
^{13}C NMR spectra of KPU-406 (125 MHz, DMSO-d_6)

Supplementary Notes

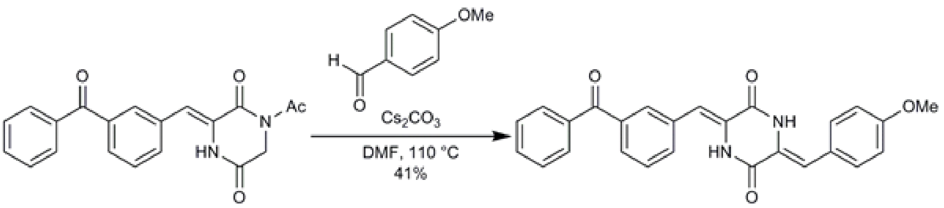

Supplementary Note 1. Synthetic scheme of (3*Z*,6*Z*)-3-(3-benzoylbenzylidene)-6-(4methoxybenzylidene)piperazine-2,5-dione (KPU-406)

To a solution of (*Z*)-*N*-acetyl-3-(3-benzoylbenzylidene)piperazine-2,5-dione (30 mg, 0.086 mmol, synthesized as described previously[1]. in DMF (2.0 ml) was added 4-methoxybenzaldehyde (14 mg, 0.10 mmol). The solution was repeatedly evacuated over a short period of time to remove oxygen. The solution was then flushed with Ar. Cs_2CO_3 was then added (42 mg, 0.13 mmol) and the evacuation–flushing process was repeated again. The resultant mixture was stirred at 110°C for 2 h, and the progression of the reaction was monitored by TLC. After the reaction had finished, the solvent was removed by evaporation and the residue was dissolved in $CHCl_3$, washed with 10% $NaHCO_3$ and brine, dried over Na_2SO_4, and concentrated *in vacuo*. The product was purified by flush column chromatography using $CHCl_3$-MeOH as an eluent to give a yellow solid (15 mg, 41%); ^{1}H NMR (500 MHz, DMSO-d_6) δ 10.34 (br s, 2H), 7.86 (s, 1H), 7.84–7.80 (m, 2H), 7.78 (d, *J* = 7.6 Hz, 1H), 7.70 (tt, *J* = 7.4, 1.3 Hz, 1H), 7.65 (ddd, *J* = 7.7, 1.3, 1.3 Hz, 1H), 7.61–7.56 (m, 3H) , 7.52 (ddd, *J* = 8.7, 3.0, 1.9 Hz, 2H), 6.99 (ddd, *J* = 8.9, 3.0, 1.9 Hz, 2H), 6.81 (s, 1H), 6.75 (s, 1H), 3.80 (s, 3H); ^{13}C NMR (125 MHz, DMSO-d_6) δ 195.5, 159.2, 158.4, 157.8, 137.3, 136.8, 133.6, 133.3, 132.8, 131.0, 130.2, 129.8, 128.84, 128.83, 128.6, 127.9, 125.5, 124.8, 115.4, 114.2, 113.3, 55.2; HRESIMS (m/z): $[M+H]^+$ calcd. for $C_{26}H_{21}N_2O_4$, 425.1501; found 425.1493.

Column chromatography was performed on silica gel 60N (Kanto Chemical Co., Inc., spherical natural, 40-50 µm). TLC was performed using Merck Silica gel 60F254 precoated plates. Proton (^{1}H) and carbon (^{13}C) NMR spectra were recorded on a Bruker DPX-500 spectrometer operating at 500 MHz for proton and 125 MHz for carbon. Chemical shifts were recorded as δ values in parts per million (ppm) and referenced according to trimethylsilane (0.00) and DMSO-d_6 (39.52) for proton and carbon NMR spectra, respectively. Mass spectrum was obtained on Waters MICRO MASS LCT–premier.

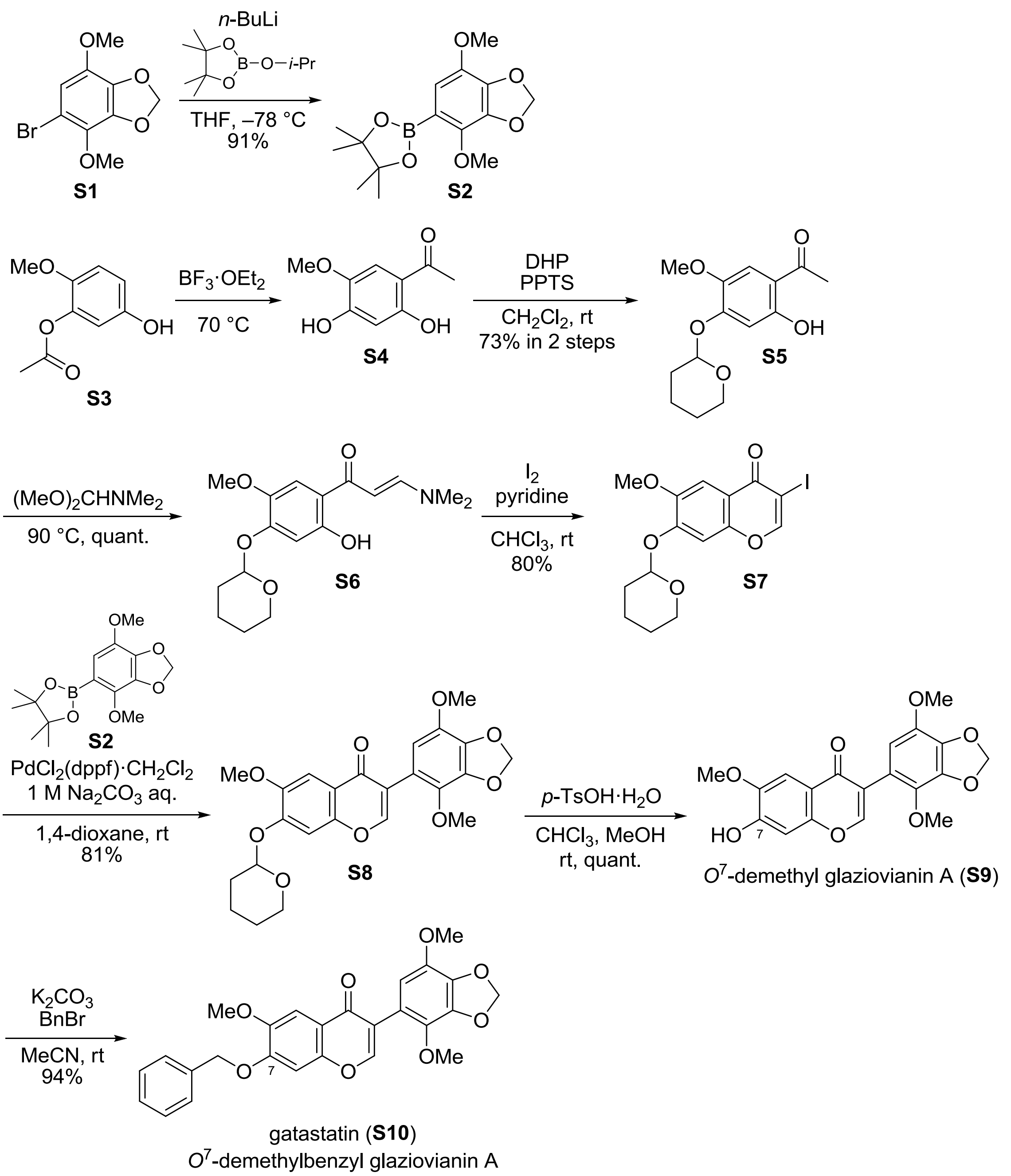

Supplementary Note 2. New synthetic scheme of 7-(Benzyloxy)-3-(4,7-dimethoxybenzo[*d*][1,3]dioxol-5-yl)-6-methoxy-4H-chromen-4-one (gatastatin, *O*^{7}-demethylbenzyl glaziovianin A)

In 2012, we synthesized *O*^{7}-demethyl glaziovianin A (**S9**) and gatastatin (*O*^{7}-demethylbenzyl glaziovianin A, **S10**) by the other synthetic method[2]. However, this synthetic method generated inseparable by-products. In this paper, we prepared *O*^{7}-demethyl glaziovianin A (**S9**) by the modified synthetic method[3]. Detailed experimental procedures and spectroscopic data of *O*^{7}-demethyl glaziovianin A (**S9**) are shown in reference 3.

Benzylation of the hydroxy group of **S9** (K_2CO_3, BnBr (benzyl bromide), MeCN (acetonitrile), r.t.) afforded gatastatin (**S10**). All the chemical shift data of gatastatin are identical with those of compound **28** in reference 2. Detailed new synthetic procedure and spectroscopic data of gatastatin (**S10**) is described as below.

Synthesis of gatastatin (**S10**): *7-(Benzyloxy)-3-(4,7-dimethoxybenzo[d][1,3]dioxol-5-yl)-6-methoxy-4H-chromen-4-one.* To a stirred solution of *O*^{7}-demethyl glaziovianin A (**S9**) (12.1 mg, 32.5 μmol) in

MeCN (2.0 ml) were added K_2CO_3 (14.0 mg, 102 μmol) and benzyl bromide (12.0 μl, 101 μmol) at room temperature. After being stirred at room temperature for 22 h, the mixture was diluted with saturated aqueous $NaHCO_3$ (2 ml) and extracted with $CHCl_3$ (10 ml × 3). The combined extracts were washed with brine (30 ml), dried over Na_2SO_4, filtered, and concentrated. The residual solid was purified by column chromatography on silica gel (700 mg, hexane–EtOAc = 3:1→$CHCl_3$) to give a yellow amorphous (containing Pd-metal) (17.0 mg). This yellow amorphous was dissolved in $CHCl_3$ (2.0 mL), and SiliaMetS® Thiourea (25.0 mg) was added. The resulting mixture was stirred at room temperature for 17 h, filtered, and concentrated. The residual oil was purified by recycle HPLC [JAIGEL-1H-40 (600×20 mm) and JAIGEL-2H-40 (600×20 mm); flow rate 3.8 ml/min; detection, UV 254 nm; solvent $CHCl_3$] to give gatastatin (**S10**) (14.1 mg, 94%) as a yellow amorphous: ^{1}H NMR (400 MHz, $CDCl_3$) δ 7.87 (s, 1H), 7.63 (s, 1H), 7.48–7.33 (m, 5H), 6.90 (s, 1H), 6.52 (s, 1H), 6.02 (s, 2H), 5.27 (s, 2H), 3.99 (s, 3H), 3.87 (s, 3H), 3.84 (s, 3H); ^{13}C NMR (100 MHz, $CDCl_3$) δ 175.4, 153.5, 153.3, 152.1, 148.1, 139.1, 139.0, 137.1, 136.8, 135.6, 128.8 (2C), 128.3, 127.2 (2C), 121.6, 118.1, 118.0, 110.1, 105.2, 101.8, 101.4, 71.1, 60.2, 56.9, 56.4; IR ($CHCl_3$) 3008, 1607, 1501, 1470, 1298, 1227, 1153 cm^{-1}; HRESIMS (*m*/*z*): $[M+Na]^+$ calcd. for $C_{26}H_{22}NaO_8$, 485.1206; found 485.1229.

Column chromatography was performed on BW-820 MH (Fuji Silysia Chemical Ltd., 75-200 μm). TLC was performed using Merck Silica gel 60F254 precoated plates. Proton (^{1}H) and carbon (^{13}C) NMR spectra were recorded on a Bruker DPX 400 spectrometer operating at 400 MHz for proton and 100 MHz for carbon. Chemical shifts were recorded as δ values in parts per million (ppm) and referenced to the solvent peaks (δ_H = 7.26 ppm and δ_C = 77.0 ppm in $CDCl_3$). IR spectra were recorded on a JASCO FT/IR-4100 instrument and are reported in wavenumbers (cm^{-1}). Mass spectrum was obtained on Jeol AccuTOFCS JMS-T100CS spectrometer.

Supplementary References

1 Hayashi, Y. *et al.* Development of a new benzophenone-diketopiperazine-type potent antimicrotubule agent possessing a 2-pyridine structure. *ACS Med Chem Lett* **5**, 1094-1098 (2014).

2 Hayakawa, I., Ikedo, A., Chinen, T., Usui, T. & Kigoshi, H. Design, synthesis, and biological evaluation of the analogues of glaziovianin A, a potent antitumor isoflavone. *Bioorg Med Chem* **20**, 5745-5756 (2012).

3 Hayakawa, I., Shioda, S., Ikedo, A. & Kigoshi, H. Practical synthesis of glaziovianin A, a cytotoxic isoflavone, and its O^7-propargyl analogue. *Bull Chem Soc Jpn* **87**, 544-549 (2014).
